# Genome sequencing of four *Aureobasidium pullulans* varieties: biotechnological potential, stress tolerance, and description of new species

**DOI:** 10.1186/1471-2164-15-549

**Published:** 2014-07-01

**Authors:** Cene Gostinčar, Robin A Ohm, Tina Kogej, Silva Sonjak, Martina Turk, Janja Zajc, Polona Zalar, Martin Grube, Hui Sun, James Han, Aditi Sharma, Jennifer Chiniquy, Chew Yee Ngan, Anna Lipzen, Kerrie Barry, Igor V Grigoriev, Nina Gunde-Cimerman

**Affiliations:** Department of Biology, Biotechnical Faculty, University of Ljubljana, Večna pot 111, Ljubljana, SI 1000 Slovenia; National Institute of Biology, Večna pot 111, Ljubljana, SI 1000 Slovenia; US Department of Energy Joint Genome Institute, 2800 Michell Drive, Walnut Creek, CA 94598 USA; Institute of Plant Sciences, Karl-Franzens-University Graz, Holteigasse 6, Graz, A-8010 Austria; Centre of Excellence for Integrated Approaches in Chemistry and Biology of Proteins (CIPKeBiP), Jamova 39, Ljubljana, SI 1000 Slovenia

**Keywords:** *Aureobasidium pullulans*, Dothideomycetes, Genome, Stress, Haloadaptation, Halotolerance, Polyextremotolerant, New species, Opportunistic mycosis, Pullulan

## Abstract

**Background:**

*Aureobasidium pullulans* is a black-yeast-like fungus used for production of the polysaccharide pullulan and the antimycotic aureobasidin A, and as a biocontrol agent in agriculture. It can cause opportunistic human infections, and it inhabits various extreme environments. To promote the understanding of these traits, we performed *de-novo* genome sequencing of the four varieties of *A. pullulans.*

**Results:**

The 25.43-29.62 Mb genomes of these four varieties of *A. pullulans* encode between 10266 and 11866 predicted proteins. Their genomes encode most of the enzyme families involved in degradation of plant material and many sugar transporters, and they have genes possibly associated with degradation of plastic and aromatic compounds. Proteins believed to be involved in the synthesis of pullulan and siderophores, but not of aureobasidin A, are predicted. Putative stress-tolerance genes include several aquaporins and aquaglyceroporins, large numbers of alkali-metal cation transporters, genes for the synthesis of compatible solutes and melanin, all of the components of the high-osmolarity glycerol pathway, and bacteriorhodopsin-like proteins. All of these genomes contain a homothallic mating-type locus.

**Conclusions:**

The differences between these four varieties of *A. pullulans* are large enough to justify their redefinition as separate species: *A. pullulans*, *A. melanogenum*, *A. subglaciale* and *A. namibiae*. The redundancy observed in several gene families can be linked to the nutritional versatility of these species and their particular stress tolerance. The availability of the genome sequences of the four *Aureobasidium* species should improve their biotechnological exploitation and promote our understanding of their stress-tolerance mechanisms, diverse lifestyles, and pathogenic potential.

**Electronic supplementary material:**

The online version of this article (doi:10.1186/1471-2164-15-549) contains supplementary material, which is available to authorized users.

## Background

*Aureobasidium pullulans* (de Bary) G. Arnaud is a polyextremotolerant black yeast of considerable biotechnological importance, and it has exceptional stress tolerance and increasing medical relevance [1]. *A. pullulans* is well known for its production of pullulan, a neutral polysaccharide of repeating maltotriose units, which has numerous applications in medicine, pharmacy, the food industry, and other fields [2, 3]. *A. pullulans* also produces a β-glucan that shows high reactivity to human IgG antibodies [4] and possible beneficial immunomodulatory effects [5]. *A. pullulans* has an unusually large spectrum of extracellular enzymatic activities [6, 7]. Several of these are of biotechnological interest, and include: amylases, cellulases, lipases, proteases, xylanases, β-fructofuranosidases, maltosyltransferases, mannanases, and laccases (for review, see [8]). At least some of these appear to have interesting traits that are different from their homologues in other species [8, 9].

A strain of *A. pullulans* is used for the production of a cyclic peptide that has specific antifungal activity: aureobasidin A [10]. Due to its strong antagonistic activity against other microorganisms, *A. pullulans* is used as a biocontrol agent in agriculture [11]. Additionally, a recent study reported that some strains of *A. pullulans* can produce an antibacterial compound, exophilin A, as well as high yields of liamocins, and heavy oils with previously unknown acylated mannitol structures, which have possible industrial applications as surfactants [12].

The occurrence of *A. pullulans* is widespread in tropical, temperate and polar areas. It is frequently found in association with diverse plants, such as in the phyllosphere [13, 14], as an epiphyte or endophyte, on stored barley grain [15], and in coconut water [16]. *A. pullulans* has also been found in numerous other habitats, some of which are particularly unusual, such as coastal hypersaline water [17, 18], glacial ice [19, 20], other polar environments [21, 22], polluted water [23], refrigerated, frozen, salt-preserved and dried foods [24, 25], various indoor habitats (e.g., bathroom surfaces [26], house dust [27], dishwashers [28], tap water [29]), the surface of human skin [30], aviation fuel tanks [31], and the surface of synthetic polymers [32] and of degrading polyurethane and PVC plastics [33]. *A. pullulans* has been reported to cause a variety of localised infections in humans, and even systemic infections, although very rarely (for review, see [34, 35]). It has been suggested that this infection potential is at least partially supported by the production of extracellular enzymes [36], and by the pronounced stress tolerance of *A. pullulans*[1].

*Aureobasidium pullulans* has evolved an exceptional tolerance for a broad range of ecological conditions. It is considered a polyextremotolerant organism [1, 37], and it can survive hypersaline [17], acidic, basic [38, 39], cold and oligotrophic [40] conditions. Some of the *A. pullulans* adaptations to stress (and especially to elevated salt concentrations) are associated with rigorous management of intracellular concentrations of alkali-metal cations [41], synthesis of compatible solutes [42] and of mycosporines [43], and adaptation at the level of the membrane-lipid composition [44, 45]. *A. pullulans* can also mitigate environmental stress by rapid dimorphic switching from small colourless yeast cells to thick-walled, heavily melanised, meristematic forms [46].

**Figure 1:**
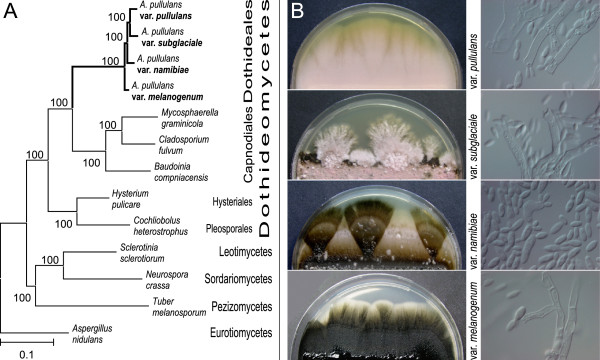
**The** ***Aureobasidium pullulans*** **varieties. A**. Phylogram showing the phylogenetic relationships of the four *A. pullulans* varieties and their phylogenetic position, inferred from super alignment of selected fungal proteomes. Chi^2^-based branch supports are shown, calculated according to the approximate Likelihood-Ratio Test, as implemented in Phyml 3.0. **B**. Representative images of one-month-old cultures of the four *A. pullulans* varieties (as indicated) on malt extract agar and microscopy images of cultures after one week of growth on malt extract agar blocks.

The genus *Aureobasidium* is a member of the order Dothideales (Ascomycota, Dothideomycetes; Figure 1), and it comprises 27 taxa (species and varieties), with *A. pullulans* being by far the most studied of these. Although the distinction of varieties and forms of *A. pullulans* has been suggested, the name *A. pullulans* is mainly used in research databases. The most recently described species of *Aureobasidium* are *A. leucospermi* Crous [47], *A. proteae* (Joanne E. Taylor & Crous) Joanne E. Taylor & Crous 2011 [47], and *A. thailandense* S.W. Peterson, Manitchotpisit & Leathers [48].

Various loci have been sequenced in the past to infer the taxonomy and phylogeny of the taxa in *Aureobasidium,* such as internal transcribed spacer (ITS) rDNA, intergenic spacer 1, translation elongation factor-1α, β-tubulin, and RNA polymerase II [19, 47–49]. Based on a multilocus analysis of a worldwide selection of *A. pullulans*-like isolates, it was confirmed that the earlier described variety *A. pullulans* var. *melanogenum* Hermanides-Nijhov is distinct from *A. pullulans* (de Bary) G. Arnaud 1918. In addition, two new varieties have been described: *A. pullulans* var. *subglaciale* Zalar, de Hoog & Gunde-Cimerman, and *A. pullulans* var. *namibiae* Zalar, de Hoog & Gunde-Cimerman. *A. pullulans* var. *aubasidani* Yurlova (Yurlova and de Hoog), which had been previously described due to its structurally unique polysaccharide, aubasidan, was synonymised with *A. pullulans* var. *pullulans*[19].

In 2008, the infraspecies classification of the varieties was retained in the redefinition of the species to prevent confusion linked to the use of new or additional epithets in applied fields [19]. Nevertheless, substantial phenotypic differences in growth-temperature range, melanisation, and tolerance to NaCl had already been observed among the varieties. *A. pullulans* var. *pullulans* is found mostly in mildly osmotic environments, it is frequently associated with plants, and it can tolerate up to 17% NaCl (w/v), the highest salt concentration of all four of the *A. pullulans* varieties [19]. *A. pullulans* var. *melanogenum* has been isolated mainly from oligotrophic, aqueous environments, and it grows at 37°C, while the other varieties cannot. On the other hand, *A. pullulans* var. *subglaciale* is unique for its psychrotolerant nature (it grows at 4°C) and its occurrence in glacial habitats in Svalbard (Norway). Finally, *A. pullulans* var. *namibiae* was named based on a single isolate from Namib Desert marble [19]. Polymorphism is evident within *A. pullulans*, even within the varieties (Figure 1), and the species has been proposed as a model for the investigation of fungal phenotypic plasticity, because of the unique colony morphologies, the frequent changes in appearance and growth-temperature regimes, and the different nutrient requirements and morphological responses to light among its strains and varieties [50].

The genome sequence of one strain of each of the above-described varieties of *A. pullulans* was obtained: *A. pullulans* var. *pullulans*, *A. pullulans* var. *melanogenum*, *A. pullulans* var. *subglaciale* (type strain), and *A. pullulans* var. *namibiae* (type strain). The results of the genome analyses performed are presented and discussed in the following sections.

## Results and discussion

The sequencing of the genomes of the four varieties of *A. pullulans* was aimed to uncover the genetic basis of their many interesting and useful traits. This included three aspects in particular: (i) their biotechnological potential (e.g., genes for extracellular enzymes, and for production of pullulan, aureobasidin A, and other compounds of interest); (ii) their exceptional stress tolerance (mainly focusing on genes associated with the synthesis of compatible solutes and water management, transport of alkali-metal ions and water, the high osmolarity glycerol [HOG] pathway, and melanin synthesis); and (iii) evidence in favour of describing these varieties as separate species.

### Genome properties: sequencing and assembly of the genomes

The genome assembly sizes of the four sequenced varieties of *A. pullulans* were 29.62 Mb (var. *pullulans*), 25.80 Mb (var. *subglaciale*), 25.43 Mb (var. *namibiae*), and 26.20 Mb (var. *melanogenum*) (Table 1). These are smaller than the sizes of all but one of the 18 Dothideomycete fungi that were compared by Ohm et al. [51], and that had an average genome size of almost 39 Mb.

**Table 1:** **Genome assembly and annotation statistics for the four** ***Aureobasidium pullulans*** **varieties**

Statistic	Value per ***A. pullulans*** variety*			
	ApP	ApS	ApN	ApM
**Assembly statistics**				
Assembly length (Mbp)	29.62	25.80	25.43	26.20
Contig length total (Mbp)	29.59	25.79	25.43	26.20
Number of contigs	209	84	55	174
Contig N50	11	12	10	12
Contig L50 (kbp)	779.84	805.54	1053.18	652.37
Number of scaffolds	186	75	47	150
Scaffold N50	10	11	9	10
Scaffold L50 (Mbp)	1.17	0.82	1.07	0.82
Number of scaffold gaps	23	9	8	24
Scaffolds gaps length (bp)	35277	5530	800	3572
Percentage of scaffolds in gaps (%)	0.12	0.02	0.00	0.01
Number of repeat-covered regions	2593	1835	1964	1836
Length of repeat-covered regions (bp)	428704	224724	197767	255194
Percentage assembly covered by repeats (%)	1.45	0.87	0.78	0.97
GC content (%)	50.02	50.78	51.12	49.85
**Gene statistics**				
Number of genes	11866	10809	10266	10594
Protein length (amino acids, median)	369	377	380	372
Exon length (bp, median)	369	363	288	294
Gene length (bp, median)	1399	1424	1266	1232
Transcript length (bp, median)	1297	1325	1140	1116
Intron length (bp, median)	57	57	57	57
Number of genes with intron	8407	7819	7632	7797
Percentage of genes with an intron	70.85%	72.34%	74.34%	73.60%
Introns per gapped gene (median)	2	2	2	2
Intergenic distance (bp, median)	681	658	699	695
GC content of exons (%)	52.52	52.53	52.99	51.87
**Functional annotations**				
Genes with KEGG annotation [n, (%)]	9322 (78.56)	8798 (81.40)	8745 (85.18)	8855 (83.59)
Genes with KOG annotation [n, (%)]	7620 (64.22)	7253 (67.10)	7165 (69.79)	7245 (68.39)
Genes with Swissprot hit [n, (%)]	7909 (66.65)	7516 (69.53)	7465 (72.72)	7541 (71.18)
Genes with Pfam domain [n, (%)]	6613 (55.73)	6305 (58.33)	6203 (60.42)	6283 (59.31)
Genes with transmembrane domain [n, (%)]	2333 (19.66)	2140 (19.80)	1984 (19.33)	2021 (19.08)

*ApP, *A. pullulans* var. *pullulans*; ApS, *A. pullulans* var. *subglaciale*; ApN, *A. pullulans* var. *namibiae*; ApM, *A. pullulans* var. *melanogenum*.

*Aureobasidium pullulans* var. *pullulans* contains more repetitive sequences than the other *A. pullulans* varieties. However, the level of the repetitive sequences is low across all four of these varieties (0.78%-1.45%), and cannot explain the differences in their genome sizes. *A. pullulans* var. *pullulans* is the most halotolerant of the four investigated varieties [19], and it was isolated from hypersaline water. Therefore, it is interesting to note that investigations of *Saccharomyces cerevisiae* have revealed a tendency towards increased genome size as a response to high concentrations of salt [52, 53]. An increased genome size was also observed for the extremely halotolerant black yeast *Hortaea werneckii*, which has experienced recent whole-genome duplication [54]. However, the increase in the genome size of *A. pullulans* var. *pullulans* is less extensive, as its genome is only 14.8% larger than the genome of its closest relative, *A. pullulans* var. *subglaciale.*

**Figure 2:**
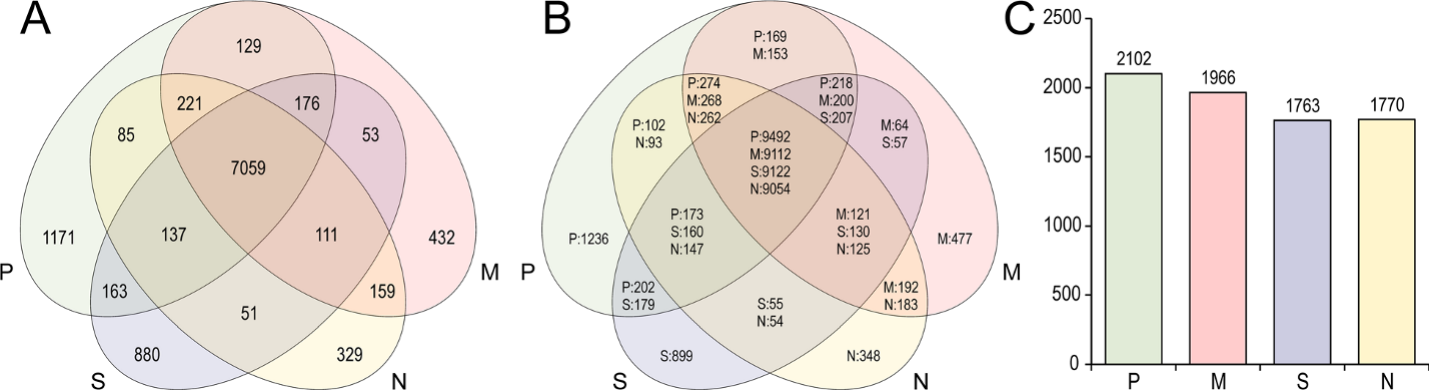
**The shared, unique and duplicated proteins of the** ***Aureobasidium pullulans*** **varieties. A**. Unique and shared protein families, as determined by the Markov clustering algorithm. **B**. Proteins shared between individual varieties, as determined by all-against-all blastp. **C**. Proteins present in at least two copies in the proteome of each of the four *A. pullulans* varieties. P, *A. pullulans* var. *pullulans*; S, *A. pullulans* var. *subglaciale*; N, *A. pullulans* var. *namibiae*; M, *A. pullulans* var. *melanogenum*.

The numbers of predicted proteins (from 10266 in *A. pullulans* var. *namibiae*, to 11866 in *A. pullulans* var. *pullulans*; Table 1) do not differ substantially from those observed in related fungi, where the average number of the predicted proteins in 18 related species that were analysed in a recent study was 11955 [51]. The median number of introns in the predicted genes of *A. pullulans* varieties is two introns per gene, with a median length of 57 bp (Table 1). Between 80% and 90% of the predicted proteins are in all four of the *A. pullulans* varieties, while 16% to 19% of the proteins are duplicated (Figure 2).

### (i) Biotechnological potential of the *Aureobasidium pullulans*varieties

#### Synthesis of pullulan

Pullulan is a linear α-D-glucan that is made of maltotriose units connected with α-1,6 linkages, while the glucose units within the maltotriose are connected with α-1,4-glycosidic bonds. This alternation of the bonds gives the molecule its distinctive physical properties: flexibility, solubility, adhesive ability, biodegradability, and the ability to form viscous solutions, and oxygen-impermeable and transparent fibres and films. Pullulan thus has broad application value [8]. Despite its economic importance, relatively little is known about the biosynthesis of pullulan. Duan et al. [55] proposed a biosynthetic pathway in which the key enzymes for converting glucose units into pullulan were α-phosphoglucose mutase, uridine diphosphoglucose pyrophosphorylase, and glucosyltransferase.

There are single-copy genes for phosphoglucose mutase and uridine diphosphoglucose pyrophosphorylase in all four *A. pullulans* varieties. This is expected, as both of these enzymes catalyse important metabolic reactions, and they have been evolutionarily conserved. The enzyme phosphoglucose mutase catalyses a key step in hexose metabolism: the interconversion of glucose 1-phosphate and glucose 6-phosphate. The enzyme uridine diphosphoglucose pyrophosphorylase catalyses the reversible formation of uridine diphosphoglucose from glucose 1-phosphate and UTP. As described below, the predicted *A. pullulans* secretomes contain representatives of several Carbohydrate-Active enZYme (CAZy) database families [56], which include members with glucosyltransferase activities. Thus all four *A. pullulans* varieties contain all of the putative enzymes that were proposed to be involved in the biosynthesis of pullulan by Duan et al. [55].

Kang et al. [57] showed that disruption of the putative pullulan synthetase gene (*pul*) of *A. pullulans* [GenBank:AF470619] reduces its exopolysaccharide production to a pure β-glucan. Each of the predicted proteomes of the four *A. pullulans* varieties contains one similar protein (JGI Protein IDs: 349889, 3372, 53761, 64747, in *A. pullulans* var. *pullulans*, *subglaciale*, *namibiae*, and *melanogenum*, respectively; e-values between 10^-22^ and 10^-27^). All of these are predicted as being secreted. The low similarity scores can be attributed to the differences in annotation, as the *pul* gene prediction in GenBank contains an atypically long intron (almost 600 bp) and its second exon lies in a poorly conserved region (Additional file 1). When the annotation of the *pul* gene [GenBank:AF470619] was corrected to correspond to our annotation, it contained a one-nucleotide frameshift–insertion at nucleotide position 62 (Additional file 1, blue). After this was removed, the resulting protein-coding sequences and the predicted protein sequences were more than 70% identical. It is possible that the insertion in AF470619 was an artefact of the sequencing and that it led to the differences in the above-described gene annotation. If the *pul* gene is indeed involved in pullulan production, this observation is of significant importance.

#### Synthesis of aureobasidin A and siderophores

Strains of *A. pullulans* are used efficiently as biocontrol agents of post-harvest diseases [11]. Not only numerous hydrolytic enzymes [58], but also various antimicrobial compounds, such as exophilin A [12], siderophores [59] and aureobasidin A [10], might have roles in the strong antagonistic effects of *A. pullulans* towards other species.

The antibiotic aureobasidin A is a cyclic nonadepsipeptide [10] that shows strong fungicidal activity, including against *Candida* species, *Cryptococcus neoformans*, and some *Aspergillus* species [60]. Aureobasidin A has been shown to inhibit the phosphatidylinositol:ceramide phosphoinositol transferase that is involved in sphingolipid synthesis [60]. The synthesis of aureobasidin A is catalysed by the 11659-amino-acid-long biosynthesis complex that is encoded by the intronless gene *aba1* of the *A. pullulans* strain R106 [61].

The four sequenced *A. pullulans* varieties have not been tested previously for aureobasidin A production. However, their genomes do not include any homologues of the *aba1* gene. This is not entirely surprising, as the production of aureobasidin A is not a universal trait of *A. pullulans*.

However, in a search for aureobasidin A synthase, a group of similar non-ribosomal peptide synthases was identified, although these proteins are substantially shorter than aureobasidin A synthase (1123–2920 amino acids) and are most similar to synthases for siderophores. *A. pullulans* var. *melanogenum* and *A. pullulans* var. *namibiae* have one putative synthase for siderophores, whereas *A. pullulans* var. *subglaciale* has two copies, and *A. pullulans* var. *pullulans* has three.

Siderophores are iron-chelating compounds with substantial biotechnological potential that are known to be produced by *A. pullulans*[62]. Siderophores have been reported to act as antimicrobials [59]. Their presence is also in line with the oligotrophic nature of *A. pullulans*, as they are beneficial under conditions of iron-depletion. Additionally, in other fungi, siderophores have roles in virulence, fungal–host interactions, and resistance to oxidative stress [63].

#### Secondary metabolite biosynthesis clusters

Fungi produce a multitude of low-molecular-mass compounds, known as secondary metabolites, which have roles in a range of cellular processes, such as transcription, development, and intercellular communication [64]. Many of these compounds have important applications as antibiotics or immunosuppressants [64], and they are used in medicine or for plant protection [65]. Genome mining investigations have indicated that the ability of fungi to produce secondary metabolites has been substantially underestimated, because many of the fungal secondary metabolite biosynthesis gene clusters are not expressed under standard cultivation conditions [64].

In *A. pullulans* var. *pullulans*, only nine secondary metabolite biosynthetic clusters were identified with Antibiotics and Secondary Metabolites Analysis Shell (antiSMASH 2.0; http://antismash.secondarymetabolites.org/). However, there are 32 clusters in *A. pullulans* var. *namibiae*, and 37 in each of *A. pullulans* var. *melanogenum* and *A. pullulans* var. *subglaciale* (Table 2; Additional file 2). These cluster numbers are surprisingly high, and although many cannot be assigned to production of a certain metabolite, their abundance suggests that the genomes of these *A. pullulans* varieties represent rich resources for secondary metabolite biosynthesis. In comparison, antiSMASH predicted 45 T1 polyketide synthase/non-ribosomal peptide synthase/*dmat* gene clusters in *Penicillium chrysogenum*, 29 clusters in *Aspergillus fumigatus*[66], and a total of 35 putative gene clusters in a draft genome sequence of *Streptomyces ansochromogenes* (Streptomycetes are known for their complex secondary metabolism) [67]. For example, lantipeptide biosynthetic clusters from *A. pullulans* var. *pullulans*, var. *namibiae* and var. *melanogenum* might be of particular interest. Lantipeptides are post-translationally modified peptides that have antimicrobial properties. Lantipeptides were initially believed to be produced only by Gram-positive bacteria, but later studies revealed a much wider diversity of lantipeptide producers than previously appreciated (and also of their activity, structure, and biosynthetic machinery) [68]. Although dozens of new lantipeptides have been isolated in recent years, bioinformatic analyses indicate that many hundreds more await discovery, owing to the widespread frequency of the lantipeptide biosynthetic machinery in the bacterial genome [68].

**Table 2:** **Secondary metabolite biosynthetic clusters for the four** ***Aureobasidium pullulans*** **varieties**

Secondary metabolite biosynthetic cluster	Number per ***A. pullulans*** variety*			
	ApP	ApS	ApN	ApM
Terpene	1	3	3	5
Lantipeptide	1	0	1	1
Type I PKS	1	6	4	4
Type III PKS	0	0	1	1
NRPS	3	2	2	2
Other	3	7	7	5
Putative	0	14	14	19
Terpene-t1pks	0	1	0	0
NRPS-t1pks	0	2	0	0
T1pks-NRPS	0	1	0	0
**Total number of clusters**	**9**	**37**	**32**	**37**

*ApP, *A. pullulans* var. *pullulans*; ApS, *A. pullulans* var. *subglaciale*; ApN, *A. pullulans* var. *namibiae*; ApM, *A. pullulans* var. *melanogenum*; PKS, polyketide synthase; NRPS, non-ribosomal peptide synthases.

#### Secreted proteins

An unusually large spectrum of extracellular enzymatic activities has been described for *A. pullulans*, many of which are of considerable biotechnological interest [6–8]. Some of these, such as alkaline serine proteases, glucanases and chitinases, are also believed to have roles in the above-described antagonistic effects of *A. pullulans* against phytopathogenic fungi [58].

The *in-silico* predicted set of secreted proteins comprises 869 proteins for *A. pullulans* var. *pullulans* (7.3% of the predicted proteome), 813 for *A. pullulans* var *subglaciale* (7.5%), 734 for *A. pullulans* var. *namibiae* (7.1%), and 725 for *A. pullulans* var. *melanogenum* (6.8%) (Table 3 and Additional file 3). The proportion of the proteins that contain signalP (predictor for secretory signal peptides) and TMHMM (predictor for transmembrane helices) sequences is comparable to that in other fungi [69]. The largest group of the secretory proteins are those similar to enzymes that are active against carbohydrates (41.1% to 45.8% of total secreted proteins; Table 3). Most of these are glycoside hydrolases (GHs; approximately a quarter of the secretome, and over half of the enzymes that are active against carbohydrates).

**Table 3:** **Predicted secreted proteins of the four** ***Aureobasidium pullulans*** **varieties**

Predicted secreted proteins	Number per ***A. pullulans*** variety*				
	ApP	ApS	ApN	ApM	All
**Total**	**869**	**813**	**734**	**725**	**3141**
**Carbohydrate-active enzymes**	**367**	**334**	**336**	**305**	**1342**
Glycoside hydrolases	207	191	186	180	**764**
Carbohydrate esterases	47	42	39	35	**163**
Glycosyltransferases	10	11	11	11	**43**
Polysaccharide lyases	11	11	10	4	**36**
Auxiliary activity	33	31	39	31	**134**
Carbohydrate-binding module	59	48	51	44	**202**
**Peptidases**	**72**	**60**	**59**	**61**	**252**
Serine peptidases	49	40	39	39	**167**
Aspartic peptidases	10	8	9	9	**36**
Metallo peptidases	8	7	7	7	**29**
Glutamic peptidases	3	2	2	3	**10**
Cysteine peptidases	1	2	1	2	**6**
Threonine peptidases	1	1	1	1	**4**
**Lipases**	8	11	8	7	**34**
**Peroxidases**	5	7	7	7	**26**
**Other functions**	90	88	100	90	**368**
**Unknown function**	327	313	224	255	**1119**

*ApP, *A. pullulans* var. *pullulans*; ApS, *A. pullulans* var. *subglaciale*; ApN, *A. pullulans* var. *namibiae*; ApM, *A. pullulans* var. *melanogenum*.

Large enzyme groups are written in bold text. Their subgroups are written in normal text.

All four of these *A. pullulans* varieties also contain a large number of enzyme families, the members of which are known to be involved in the degradation of plant material, as designated by [70]. These are discussed below.

The enzymes involved in the degradation of pectin are represented by three families of polysaccharide lyases (PL1, PL3, PL4), and the families of GH28 (polygalacturonases), GH78 (rhamnosidases), GH105 (rhamnogalacturonyl hydrolases) and the carbohydrate esterases CE8 (pectinesterases). The GH88 family (d-4,5-unsaturated β-glucuronyl hydrolases) is only present in *A. pullulans* var. *namibiae*, although it is not recognised as secreted. No representatives of the polysaccharide lyases from the PL9 and PL11 families were identified.

In all four of these *A. pullulans* varieties we identified five out of seven protein families with members that are involved in (among other activities) the degradation of cellulose (they contain the GH6, GH7, GH12, GH45, and AA9 families, but not the GH74 and GH94 families). *A. pullulans* var. *namibiae* contains all of the 15 families that contain hemicellulases (GH10, GH11, GH27, GH29, GH35, GH36, GH39, GH43, GH51, GH53, GH54, GH62, GH67, GH93, GH115), while the other three *A. pullulans* varieties lack the GH39 family (Additional file 3). This diversity is comparable to, and in some cases even larger than, other plant pathogenic fungi [70], including those from Dothideomycetes (Additional file 4) [51], and it is larger than in most of the saprophytic fungi, especially in the case of the hemicellulases (Additional file 4) [70].

Although the sequence-based families of carbohydrate-active enzymes frequently group together enzymes of varying substrate specificities, they are nevertheless considered to be good reporters of fungal lifestyles, especially when considering broad substrate categories, such as cellulose, hemicellulose or pectin [51]. The richness of the enzyme families that are involved in the degradation of plant material is thus interesting in light of the epiphytic lifestyle of *A. pullulans*. When comparing individual varieties, proteins containing PFAM protein family domains (http://pfam.sanger.ac.uk/), which are characteristic for the GH3 (PF00933, PF01915), GH5 (PF00150), GH16 (PF00722), GH28 (PF01915), and GH43 (PF04616) families, are significantly enriched in *A. pullulans* var. *pullulans* compared to the other varieties (using the Computational Analysis of Gene Family Evolution [CAFE] software [71]), while the GH3 and GH5 families are depleted in *A. pullulans* var. *melanogenum*. This corresponds to the plant-related ecology of *A. pullulans* var. *pullulans* and to the largest phylogenetic distance between *A. pullulans* var. *pullulans* and *A. pullulans* var. *melanogenum*.

The numbers of genes and families of secreted proteases in the genomes of *A. pullulans* are also comparable to those found in the dothideomycete plant pathogens [51]. All of the subfamilies of the MEROPS database of proteolytic enzymes [72], which are expected to efficiently digest proteins and/or to work in inhospitable environments of the extracellular matrix (A01, C13, G01, M35, M20, S08, S09, S10; [51]), are represented in these predicted secretomes. The only exception is C13 protease*,* which is recognised as being secreted only in *A. pullulans* var. *melanogenum*, whereas the homologues from the other varieties are not. The family of scytalidoglutamic peptidases (G01) contains even more representatives than in the analysed plant pathogens. Aspartic proteases (PF00026) and the A4 family (PF01828) are enriched in *A. pullulans* var. *pullulans*.

The number of lipases in the predicted secretomes is relatively low, although the number of cutinases, which are enzymes that are important for the degradation of the plant cuticle, is in the range observed in related plant pathogens [51] (Table 3). However, some of the putative cutinases are not designated as secreted with the criteria used in the present analysis, so the actual number of these enzymes might be even higher.

Tannases are another group of enzymes that are significantly enriched within the genome of *A. pullulans* var. *pullulans*. Tannases, or tannin acyl hydrolases (EC, 3.1.1.20), catalyse the hydrolysis of ester bonds in the hydrolysable tannins and gallic acid esters. These enzymes are used industrially as catalysts in the manufacture of gallic acid, and they also have potential use in beverage and food processing [73].

Given the previously recognised substantial biotechnological potential of the large spectrum of the extracellular enzymatic activities of this fungus [6–8], which is confirmed here by the predicted secretome, their application should be brought to a new level with the availability of these genomic data. The wide array of enzymatic activities should also be considered when investigating the pathogenicity potential of *A. pullulans*. As already noted by Chan et al. [36], lipases, phospholipases, proteases and β-lactamases are important virulence factors that might have roles in *A. pullulans* as an emerging opportunistic human pathogen.

#### Biodegradation of plastic and aromatic compounds

Black yeast, including *A. pullulans*, are known for their ability to degrade aromatic pollutants [74, 75] and plastic [76, 77]. In the *A. pullulans* AY4 strain, Chan et al. [36] reported the presence of genes coding for 2-monooxygenases and catechol dioxygenases (biodegradation of aromatic substances) and depolymerase (biodegradation of plastic). There are two families of 2-monooxygenases in these four *A. pullulans* varieties. The larger of these has six representatives in each of *A. pullulans* var. *pullulans* and *A. pullulans* var. *subglaciale,* five in *A. pullulans* var. *melanogenum*, and four in *A. pullulans* var. *namibiae.* The second family contains only one representative in *A. pullulans* var. *pullulans* and *A. pullulans* var. *subglaciale*. A family of proteins with catechol 1,2-dioxygenase activity is also present, with four representatives in *A. pullulans* var. *melanogenum* and five in each of the other three *A. pullulans* varieties. There are single-copy genes for poly-β-hydroxybutyrate depolymerase, an enzyme that can degrade polyhydroxyalkanoates [78], in all four of these *A. pullulans* varieties. The presence of these genes underlines the considerable potential of *A. pullulans* for use in bioremediation or biodegradation processes.

#### Major facilitator superfamily sugar transporters

The major facilitator superfamily (MFS) of sugar transporters is the largest group of secondary transmembrane carriers. This consists of 74 protein families, each of which is specialised for the transport of a certain type of substrate. They have different transport modes, from uniport, to solute:cation (H^+^ or Na^+^) symport and/or solute:proton or solute:solute antiport. Most of these have 400 to 600 amino acyl residues, with two six-transmembrane segment repeat units [79]. According to the Transporter Classification (TC) database [80], two representatives in this group, the MFS family (TC no. 2.1) and the glycoside-pentoside-hexuronide:cation symporter family (TC no. 2.2), contain sugar-specific families of secondary active transporters. In Eukarya, only two families of MFS sugar transport substrates are known, of which the sugar porter family (TC no. 2.1.1) is the largest of all of the MFS families, with several hundred known members. The sugar porter family is diverse with respect to their substrate specificity, and they can work either as uniporters or as cation symporters. The other familiy of sugar transporters is the sialate:H1 symporter family (TC no. 2.1.12) [81].

According to CAFE analysis, proteins that contain the PFAM domain, which is characteristic for sugar-transport proteins (PF00083), are significantly enriched in the genomes of all four *A. pullulans* varieties. When comparing these varieties individually, the sugar-transport proteins are also significantly enriched in *A. pullulans* var. *pullulans*, while they are depleted in *A. pullulans* var. *melanogenum*. Detailed analysis has revealed great diversity of these transporters (Table 4). The sugar porter family contains from 73 (*A. pullulans* var. *melanogenum*) to 92 (*A. pullulans* var. *pullulans*) predicted proteins, which is much higher compared to the 32 transporters of *Neurospora crassa*[82], or the 31 of *S. cerevisiae*[82, 83] (Table 4). However, it is lower than the number of MFS sugar transporters in *Aspergillus nidulans* (100) and *Aspergillus oryzae* (119) (http://www.membranetransport.org, 1. 8. 2013). More than three quarters of the transporters in these four *A. pullulans* varieties are classified as sugar: H^+^ symporters (Table 4), while in *S. cerevisiae*, more than half are uniporters [83].

**Table 4:** **Secondary active sugar transporters within the major facilitator superfamily (MFS) in the four** ***Aureobasidium pullulans*** **varieties**

Transporter classification (TC) no.	Transporter	Number per ***A. pullulans*** variety and yeast*				
		ApP	ApS	ApN	ApM	Sc
**2.A.1**	**Major facilitator superfamily**					
**2.A.1.1**	**Sugar porter family**	**92**	**86**	**84**	**73**	**31**
2.A.1.1.7	Quinate: H^+^ symporter or	13	15	14	12	3
2.A.1.1.8	Myo-inositol: H^+^ symporter or					
2.A.1.1.104	Myo-inositol/H^+^ symporter					
2.A.1.1.9	Lactose, galactose: H^+^ symporter or	10	10	11	7	
	Lactose permease					
2.A.1.1.10	Maltose: H^+^ symporter or	16	15	17	9	4
2.A.1.1.11	General α-glucoside: H^+^ symporter					
2.A.1.1.33	Fructose: H^+^ symporter	1	1	1	1	
2.A.1.1.38	Glycerol: H^+^ symporter	12	13	16	11	1
2.A.1.1.39	High affinity glucose transporter (probably H^+^ symporter)	2	2	2	4	
2.A.1.1.40	Xylose: H^+^ symporter	6	6	3	5	
2.A.1.1.57	High affinity glucose: H^+^ symporter (monosaccharides including xylose)	3	3	3	3	
2.A.1.1.58	Low affinity glucose: H^+^ symporter	2	2	2	2	
Unidentified	Galactose: H^+^ symporter	7	3	3	4	
Unidentified	Hexose transporter	8	4	3	4	
Unidentified	MFS monosaccharide transporter	6	6	4	5	1
Unidentified	MFS sugar transporter	2	2	2	2	
2.A.1.1.108	Low-affinity glucose transporter Hxt1/3					2
2.A.1.1.111	High-affinity glucose transporter Hxt2					1
2.A.1.1.5	Hexose uniporter Hxt 10					1
2.A.1.1.30	Low affinity, constitutive, glucose (hexose; xylose) uniporter Hxt4					1
	Hexose transporter with moderate affinity for glucose Hxt5					1
2.A.1.1.31	High affinity, glucose-repressible, glucose (hexose) uniporter Hxt6/7					2
	Hexose transporter Hxt8					1
2.A.1.1.105	Hexose transporter Hxt9/11					2
2.A.1.1.110	Hexose transporter Hxt13/17					2
2.A.1.1.109	Hexose transporter Hxt14					1
2.A.1.1.107	Hexose transporter Hxt15/16					2
2.A.1.1.6	Galactose, glucose uniporter Gal2					1
2.A.1.1.68	Glucose transporter/sensor	3	3	2	3	2
2.A.1.1.19						
2.A.1.1.18						
2.A.1.1.93	Vacuolar protein sorting-associated protein 73 or	1	1	1	1	3
2.A.1.1.96	Probable metabolite transport protein YBR241C or					
2.A.1.1.100	Probable metabolite transport protein YFL040W					
**2.A.1.12**	**The sialate: H** ^**+**^ **symporter family**	**1**	**1**	**1**	**1**	**1**
2.A.1.12.2	Lactate/pyruvate: H^+^ symporter	1	1	1	1	1
**2.A.2**	**The glycoside-pentoside-hexuronide: cation symporter family**	**2**	**1**	**1**	**2**	
2.A.2.6.1	Maltose/sucrose H^+^ symporter Sut1	2	1	1	2	
	Metabolite/H^+^ symporter					

*ApP, *A. pullulans* var. *pullulans*; ApS, *A. pullulans* var. *subglaciale*; ApN, *A. pullulans* var. *namibiae*; ApM, *A. pullulans* var. *melanogenum*; Sc, *S. cerevisiae*.

Large transporter groups are written in bold text. Their subgroups are written in normal text.

The high numbers of sugar transporters correspond to the nutritional diversity of *A. pullulans*, while the differences between the four *A. pullulans* varieties might reflect their ecological preferences. *A. pullulans* var. *pullulans* is most frequently associated with plants, and it thus has access to various sugars of plant origin. On the other hand, *A. pullulans* var. *melanogenum* has significantly fewer sugar transporters, and it is associated more with freshwater habitats, where simple sugars are not as readily available. It is also separated from *A. pullulans* var. *pullulans* by the largest evolutionary distance of all of these *A. pullulans* varieties. The most notable difference between these varieties is in the number of the putative maltose or α-glucoside: H^+^ symporters (TC no. 2.A.1.1.10 or 2.A.1.1.11), as *A. pullulans* var. *melanogenum* has almost half the number of copies compared to the other varieties.

### (ii) Polyextremotolerance

#### Components of the high-osmolarity glycerol pathway

The mitogen-activated protein kinases (MAPKs) are involved in many cellular processes, such as stress responses and regulation of differentiation and proliferation, and they are highly conserved in eukaryotes. This system consists of three kinases that phosphorylate one another in a signalling cascade. One of the best-characterised is the high-osmolarity glycerol (HOG) pathway, which is a branched MAPK signal-transduction system, the physiological role for which in fungi is primarily the mediation of cellular adaptation to increased osmolarity of the surrounding medium. The HOG pathway is also required for adaptation to other stress conditions, such as oxidative, heavy-metal, and hot or cold stress [84] and in the virulence of pathogenic fungi [85].

The HOG pathway in fungi consists of two major signalling modules. One is the MAPK module that comprises MAPK, MAPK kinase (MAPKK), and MAPKK kinase (MAPKKK). The other module is a two-component phospho-relay system that is composed of hybrid sensor kinases, a histidine-containing phosphotransfer protein, and response regulators; this system senses and relays environmental signals, and subsequently activates the HOG pathway [84, 85].

As expected, the main components [86] in the four *A. pullulans* varieties are very similar to those in other fungi. These genomes contain one homologue of each kinase in the MAPK module (Hog1 MAPK, Pbs2 MAPKK, and two MAPKKKs, Ssk2/22 and Ste11), one Ste50 kinase homologue, and one homologue of all of the components in two sensory branches Sln1 and Sho1 (Sln1, Ypd1, Ssk1, Sho1). Interestingly, *A. pullulans* var. *pullulans* and *A. pullulans* var. *namibiae* have two predicted homologues of Ste20 or Cla4 kinases (activators of Ste11 MAPKKK), while the other two varieties have only one. One of the duplicated homologues is missing the pleckstrin-homology domain (PF00169). This domain has roles in the recruitment of proteins to different cellular membranes, and thus enables the proteins to interact with other components of the signal-transduction pathways [87]. Whether the observed difference has a role in the polyextremotolerance of *A. pullulans* remains a question for future studies.

#### Genes involved in the biosynthesis of compatible solutes

Stress tolerance is frequently associated with accumulation of small organic molecules that can act as protective compounds against different stress factors (for review, see [88]). In *A. pullulans*, exposure to high temperatures or high salt concentrations, or a combination thereof, leads to increased intracellular concentrations of the polyhydroxy compounds trehalose, mannitol and glycerol [42]. The concentrations of trehalose in *A. pullulans* increase in heat-stressed cells, and in simultaneously heat-stressed and salt-stressed cells, but not in cells subjected to salt stress alone. Mannitol increases under all of the stress conditions that have been examined, while an increase in intracellular glycerol has only been detected in salt-stressed cells [42]. Indeed, in response to a saline stress, glycerol is the most abundant compatible solute (Kogej, unpublished data). In other microbes, glycerol, trehalose and other polyols are also very important for life in extremely cold environments [89]. This might also be the case in the psychrotolerant *A. pullulans*, however, the role of compatible solutes at low temperatures has not yet been studied in this species.

Glycerol is synthesised from dihydroxyacetone phosphate, a glycolytic intermediate, via two reaction steps that are catalysed by nicotinamide adenine dinucleotide (NAD)-dependent glycerol-3-phosphate dehydrogenase (Gpd) and glycerol-3-phosphatase (Gpp) [90]. Both of the genes that encode these enzymes required for glycerol biosynthesis are present in the genomes of all four of these *A. pullulans* varieties, as single-copy genes. The yeast *S. cerevisiae* and the halophilic basidiomycete *Wallemia ichthyophaga* each have two copies of Gpd [91], while the extremely halotolerant *H. werneckii* has four, two of which can be attributed to a whole-genome duplication event [54]. The predicted Gpd proteins from *A. pullulans* lack the N-terminal PTS2 sequence that is important for peroxisome localisation, a trait also noted in other halotolerant fungi [92]. It was proposed that the constant cytosolic localisation of the Gpd1 homologues is advantageous for the organisms that live in extremely saline environments [92], as the osmoprotective role of Gpd is dependent on the cytosol and nuclear fractions of this protein [93].

The biosythesis of trehalose is a two-step process in which glucose 6-phosphate and UDP-glucose are first converted to α,α-trehalose 6-phosphate by trehalose-6-phosphate synthase (Tps), and then to trehalose by trehalose-6-phosphate phosphatase (Tpp) [94]. In *Ascomycota*, mannitol is also synthesised in two steps: fructose 6-phosphate is first reduced to mannitol 1-phosphate by NAD-dependent mannitol-1-phosphate dehydrogenase (Mpd), and then dephosphorylated to mannitol, by mannitol-1-phosphate phosphatase (Mpp) [95]. One copy of trehalose-phosphatase was found in each of the four investigated *A. pullulans* varieties, and two relatively dissimilar (~30% identical amino acids) trehalose synthases were identified. Two proteins in each variety have predicted mannitol dehydrogenase activities. One is similar to the homologues that are crucial for mannitol biosynthesis in other fungi, while the other is probably involved in the first step in the catabolism of mannitol [95]. Interestingly, no homologues of known fungal mannitol-1-phosphate phosphatases were found in any of these four *A. pullulans* genomes.

In the genomes of all four of the *A. pullulans* varieties, we identified a putative D-xylose reductase (cluster 19, 48900/38638, 70467, 36428 and 36838 for *A. pullulans* var. *pullulans*, var. *subglaciale*, var. *namibiae* and var. *melanogenum*, respectively). D-xylose reductase converts xylose to xylitol, a sweetener compound. The production of xylitol is a commercially interesting process [96, 97], which has rarely been contemplated in *A. pullulans*[98]*.*

#### Melanin biosynthesis genes

The cells of *A. pullulans* produce a black pigment that has long been known to be 1,8-dihydroxynaphthalene (DHN)-melanin [99]. Melanin is a high-molecular-weight, dark brown or black pigment that can be produced by numerous fungi [100], and it has a known protective role under various stress conditions (for review, see [88]), and also under hypersaline conditions [101]. Melanin is located in the fungal cell wall, either enmeshed within the structure of the cell wall, or as its outermost layer [102]. Microbes predominantly produce melanin pigment via tyrosinases, laccases, catecholases, and the polyketide synthase pathway [103].

Dothideomycetes mostly produce DHN-melanin via the polyketide synthase pathway [104, 105]. DHN-melanin biosynthesis starts with polyketide synthase, with acetyl coenzyme A or malonyl coenzyme A as a precursor. The polyketide synthase produces 1,3,6,8-tetrahydroxynaphthalene, which is reduced by hydroxynaphthalene reductase to form scytalone. Dehydration of scytalone by scytalone dehydratase forms 1,3,8-trihydroxynaphthalene. In turn, 1,3,8-trihydroxynaphthalene reductase converts 1,3,8-trihydroxynaphthalene to vermelone, which is further dehydrated by scytalone dehydratase, to 1,8-DHN. Subsequent steps are believed to involve dimerisation of the 1,8-DHN molecules, followed by polymerisation that is catalysed by p-diphenol oxidase [106].

One melanin polyketide synthase gene was identified in each of these four *A. pullulans* varieties. This is in agreement with findings in the other DHN-melanin–producing fungi, which also have one melanin polyketide synthase [107–109]. Additionally, there are one of each of the trihydroxynaphthalene reductase-like gene and tetrahydroxynaphthalene reductase-like gene. Each of the two reductases from these four *A. pullulans* varieties falls within either a trihydroxynaphthalene-like or a tetrahydroxynaphthalene-like group, as has been observed in other melanised fungi. One of the traits not expected is the presence of two scytalone dehydratase-like genes in all of the investigated varieties except *A. pullulans* var. *melanogenum*. These duplicated genes form two distinct phylogenetic groups. Furthermore, our phylogenetic analysis of the scytalone dehydratases from these four *A. pullulans* varieties and the scytalone dehydratase sequences collected from the NCBI-NR protein database show the presence of two scytalone dehydratases in several other fungal species as well (Additional file 5). These duplicated genes form two distinct phylogenetic groups: the larger one (cluster I) contains scytalone dehydratases from known DHN-melanin–producing fungi, and proteins 67660, 42009, 345205 and 70470 (from *A. pullulans* var. *melanogenum*, var. *subglaciale*, var. *pullulans*, and var. *namibiae*, respectively). The second cluster has two subclusters: one (IIa) contains the three duplicate proteins from *A. pullulans* var. *subglaciale*, *A. pullulans* var. *pullulans* and *A. pullulans* var. *namibiae* (676013, 282919, 48527), together with both scytalone dehydratase proteins from *Fusarium*, *Botryotinia* and *Metarhizium*. The other subcluster (IIb) contains proteins that produce bluish-green pigment through the DHN-melanin pathway [110], and it has no homologues in the four *A. pullulans* varieties (Additional file 5). This is the first report of several scytalone dehydratase genes and proteins in a single fungal species. Thus, we have identified here genes that possibly encode essential components of the DHN-melanin biosynthesis pathway in all four of these *A. pullulans* varieties.

#### Aquaporins

A large family of major intrisic proteins (MIPs), which are membrane-channel proteins that are selective for the transport of water (aquaporins) or water plus glycerol (aquaglyceroporins), has been found in diverse life forms [111]. All of the aquaporins are transmembrane proteins with six transmembrane domains, and with their N-terminus and C-terminus in the cytosol. These are water channels that are 10^9^-fold faster compared to ion channels and transporters. The presence of solute-permeable aquaporins represents a new concept in terms of absorption, not only because of the rate of the process, but also because of its osmotic implications [112].

Orthodox aquaporins mediate rapid and selective fluxes of water across biological membranes, and hence they have important roles in the osmoregulation of cells and organisms. Aquaglyceroporins, on the other hand, facilitate transmembrane transport of small uncharged molecules, like polyols, urea, arsenite, and many more, thereby having roles in nutrient uptake, osmoregulation, and probably other processes [113, 114]. Petterson et al. [115] divided the fungal aquaporins into four groups: one group of orthodox aquaporins, and three groups of aquaglyceroporins (Fps1-like, Yfl054-like, and aquaglyceroporins that do not fit into these groups). A later phylogenetic analysis of 229 major intrisic fungal proteins by Xu et al. [116] also classified the aquaporin-like genes into four clusters, delineated by functionally characterised major intrisic fungal proteins: orthodox aquaporins, aquaglyceroporins, facultative fungal aquaporins, and X intrinsic proteins.

**Figure 3:**
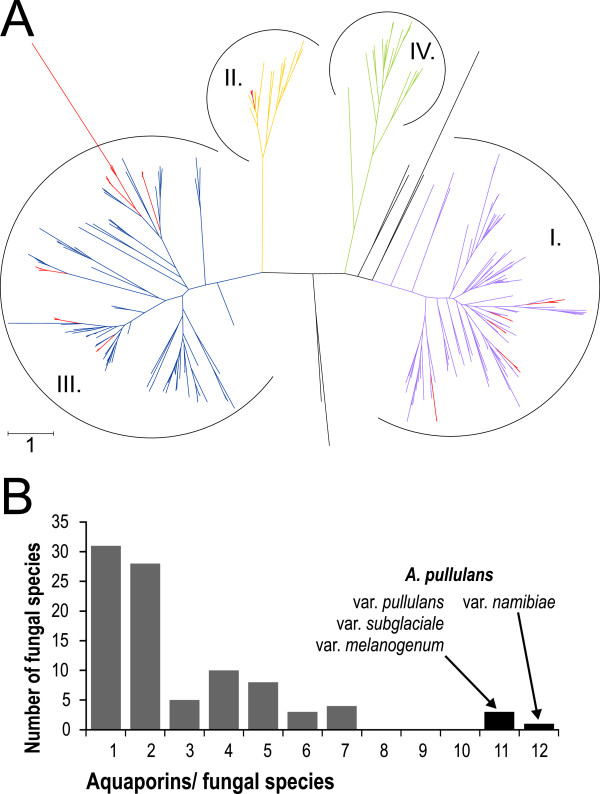
**Aquaporin genes in** ***Aureobasidium pullulans*** **and other fungi. A**. Protein tree of aquaporin-like genes from the four *A. pullulans* varieties and other fungi. The tree with GenBank accession numbers is available as Additional file 6. Colours correspond to previously recognised phylogenetic groups [116], and aquaporin-like proteins of the *A. pullulans* varieties are marked in red. **B**. Histogram showing the number of aquaporins in the four *A. pullulans* varieties, as compared to the other fungal species reported by Xu et al. [116].

Altogether, 11 aquaporin-like and aquaglyceroporin-like genes were identified in *A. pullulans* var. *pullulans, A. pullulans* var. *melanogenum* and *A. pullulans* var. *subglaciale,* and 12 in *A. pullulans* var. *namibiae*. These numbers are higher than those in other species: e.g., *Laccaria bicolor* has six or seven aquaporins, and the numbers in other investigated fungal species have been reported to be even lower [116] (Figure 3). Therefore, the high number of aquaporin-like and aquaglyceroporin-like genes in the genomes of these four polyextremotolerant *A. pullulans* varieties is striking. Such an abundance is typical for plants and mammals [116], but not for fungi. Our phylogenetic analysis of these *A. pullulans* aquaporin-like sequences and the sequences analysed by Xu et al. [116] shows that the major intrisic proteins from *Aureobasidium* varieties cluster with orthodox aquaporins, or true water channels (cluster I: five proteins in *A. pullulans* var. *namibiae*, four in each of the other varieties), with fungal aquaglyceroporins (cluster II: one protein in each of these four *A. pullulans* varieties), and with facultative aquaporins (cluster III: six proteins in each of these four *A. pullulans* varieties) (Figure 3, Additional file 6). As these water channels and osmoregulators enable sufficient water and/or compatible solute fluxes into and out of the cells under various osmotic conditions, the high abundance of aquaporins in these four *A. pullulans* varieties might be vital for survival and adaptability of these polyextremotolerant species in water-challenged environments. However, the possible beneficial effects of increased copy numbers of these proteins for survival in extreme environments have not been indicated previously. Furthermore, as only the MIPs in model yeast species and mycorrhizal fungal species have been studied so far, the aquaporins from the fungal species that inhabit various extreme habitats (e.g., drought-resistant and salt-tolerant species, like *A. pullulans* varieties) might have either high efficiency or unique mechanisms of aquaporin regulation [116] that are still awaiting discovery.

#### Alkali-metal cation transporters

As a halotolerant species, *A. pullulans* has to maintain its intracellular cation homeostasis during changing and occasionally high environmental salinities. Under such conditions, the maintenance of a high and stable K^+^ content and the elimination of toxic Na^+^ ions is crucial for survival [117, 118]. However, it is not clear if and how the number of alkali-metal cation transporter genes is correlated with the halotolerance of an organism. The genome sequencing of the extremely halotolerant black yeast *H. werneckii* revealed an extensive enrichment of genes that encode transporters of alkali-metal cations [54]. These appear to allow *H. werneckii* to maintain the very low internal Na^+^ concentrations that are observed even at high external NaCl concentrations [41]. On the other hand, genome sequencing of the most halophilic fungus known, the basidomycete *W. ichthyophaga*, uncovered only modest numbers of ion-transporter genes, and additionally showed that their transcription is relatively low and non-responsive to different salt concentrations [91].

**Figure 4:**
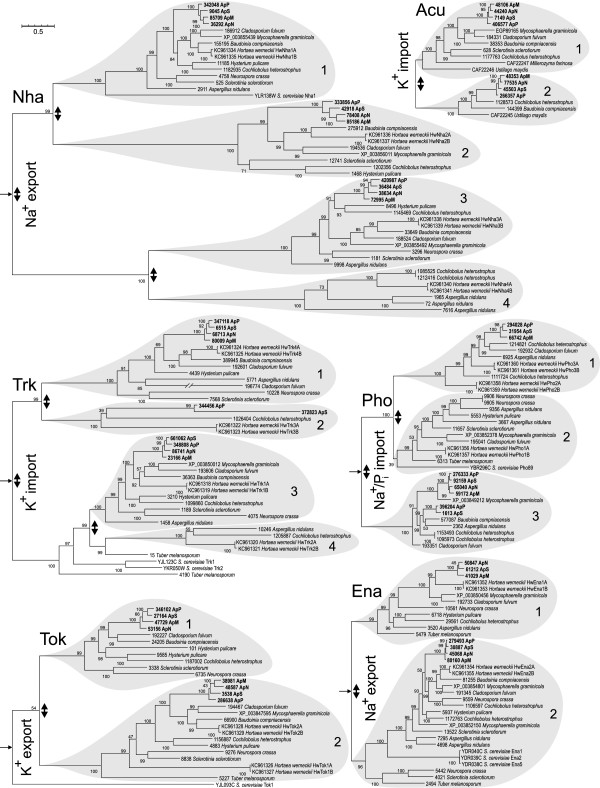
**Protein trees of the various membrane transporters of Na** ^**+**^ **and K** ^**+**^ **.** The protein trees are labelled according to the names of homologues from *S. cerevisiae* (except Acu transporters, which have no *S. cerevisiae* homologues). The trees (except for Acu) were rooted with homologous proteins from *C. neoformans* (Trk: [GenBank:XP_570017], [GenBank:XP_569339]; Tok: [GenBank:XP_568987], [GenBank:XP_568988]; Nha: [GenBank:XP_569560]; Ena: [GenBank:XP_572412], [GenBank:XP_568029], [GenBank:XP_570160]; Pho: [GenBank:XP_568082]) and root locations marked with an arrow. In addition to genes from *A. pullulans*, homologues from related fungi were used, as labelled with the fungal species name and GenBank accession number (*H. werneckii, M. graminicola, S. cerevisiae*), or the Joint Genome Institute Genome Portal protein ID (all of the rest). Putative gene duplications leading to the present diversity of these genes in *A. pullulans* are indicated by double arrows. ApP, *A. pullulans* var. *pullulans*; ApS, *A. pullulans* var. *subglaciale*; ApN, *A. pullulans* var. *namibiae*; ApM, *A. pullulans* var. *melanogenum*.

The analysis of these four *A. pullulans* genomes revealed several duplications of plasma-membrane transporters, but not of those located on the intracellular organelles (Table 5, Additional file 7). This is not entirely surprising, as the organelle membranes are protected by the relatively stable intracellular environment, rather than being exposed to the high extracellular fluctuations of inorganic salt ions [41]. From an additional analysis, which included the homologues from other Dothideomycetes along with the sequenced genomes of the four *A. pullulans* varieties, it is clear that most of the duplications observed occurred in the relatively distant evolutionary past (Figure 4). Three such events can be observed for the Nha Na^+^/H^+^ antiporter and the Trk K^+^ importer, two for the Pho Na^+^/P_i_ importer, and one for the Ena Na^+^ exporter, the Tok K^+^ exporter and the Acu K^+^ importer*. Cochliobolus heterostrophus* still has representatives in almost all of the resulting subgroups (15 of 17), followed by *A. pullulans* var*. melanogenum* (14), *A. pullulans* var*. pullulans* (13), *A. pullulans* var*. subglaciale* (13), *H. werneckii* (13), *Cladosporium fulvum* (13), and *A. pullulans* var*. namibiae* (12) (Additional file 8).

**Table 5:** **Numbers of specific types of alkali-metal cation transporters of the four** ***Aureobasidium pullulans*** **varieties**

Cell location	Transporter type	Substrate specificity/main function	Sc homologue	Number per ***A. pullulans*** variety*			
				ApP	ApS	ApN	ApM
Plasma membrane	Channel	K^+^ efflux	Tok1	2	2	2	2
	Uniporter	K^+^ uptake	Trk1,2	3	2 (+1)	2	2
	Antiporter with H^+^	K^+^ uptake (Hak)	/	1	1	1	1
	P-type ATPase	K^+^ uptake (Acu)	/	2	2	2	2
	P-type ATPase	Na^+^ (and Li^+^) efflux	Ena1,2,5	1	2	2	2
	Antiporter with H^+^	Na^+^ (and K^+^) efflux	Nha1	3	3	3	3
	Symporter	Na^+^/P_i_ cotransporter	Pho89	3	3	1	2
	P-type ATPase	H^+^ export	Pma1,2	1	1	1	1
Golgi apparatus	Antiporter	K^+^/H^+^ exchange	Kha1	1	1	1	1
	P-type ATPase	Ca^2+^ and Mn^2+^ transport into Golgi apparatus	Pmr1	2	2	2	2
Late endosomes	Antiporter	Na + (and K+) efflux	Nhx1	1	1	1	1
Vacuole	Antiporter	Na^+^, Ca^2+^/H^+^ exchange	Vnx1/Vcx1	1 (4)	1 (4)	1 (4)	1 (4)
	V-type ATPase (4 subunits)	H^+^ in vacuole	Vma1	1	1	1	1
	P-type ATPase	Depleting cytosol of Ca^2+^ ions	Pmc1	1	1	1	1
Mitochondria	Antiporter	K^+^/H^+^ exchange	Mrs7/Mdm38	1	1	1	1

*ApP, *A. pullulans* var. *pullulans*; ApS, *A. pullulans* var. *subglaciale*; ApN, *A. pullulans* var. *namibiae*; ApM, *A. pullulans* var. *melanogenum*; Sc, *S. cerevisiae*.

*S. cerevisiae* contains two typical K^+^-transport systems: Trk1 and Trk2, which are high-affinity channels for K^+^ uptake [119], while the Tok1 membrane-depolarisation-activated channel is for K^+^ efflux [120]. Ena1, Ena2 and Ena5 P-type ATPases [121–123] and the Nha1 antiporter, which is driven by a proton gradient, can also contribute to K^+^ efflux, although they are primarily known as exporters of Na^+^. In *A. pullulans*, both Trk and Tok channels are duplicated (Figure 4). *A. pullulans* var. *pullulans* contains another homologue of Trk that belongs to cluster 2, which is still present in *H. werneckii* and *C. heterostrophus*, but not in other fungi analysed. The same is true for *A. pullulans* var. *subglaciale*, although its homologue is uncharacteristically short (it contains only 137 amino acids, compared to the average of 808 for the other *A. pullulans* Trk proteins).

Unexpectedly, in addition to Trk, all of these four *A. pullulans* varieties contain two less-common K^+^ uptake systems (Table 5): the Hak H^+^/K^+^ symporters, which under some conditions appear to act as Na^+^/K^+^ symporters [124]; and the Acu P-type ATPases [124], which are encoded by not one, but two relatively divergent genes in each of these genomes (Figure 4). Both of these types of transporters are present in only a few other Dothideomycetes. According to the predicted phylogeny, it is parsimonious to assume that both of these K^+^ uptake systems were present in the ancestor of *H. werneckii* and were lost later in the evolution of this by-far-the-most halotolerant of all of the species analysed. Therefore, the primary purpose of the redundancy of these K^+^ uptake systems might not be adaptation to salt.

In other groups of transporters two duplications were observed in the above-mentioned Nha Na^+^/H^+^ antiporter, and one duplication in the Ena Na^+^ exporters (Figure 4). The smaller number of Ena genes compared to Nha (Table 5) has already been noted for *H. werneckii*[54]. Two duplication events and the subsequent loss of genes (Figure 4) led to different numbers of Pho Na^+^/P_i_ symporters in these individual *A. pullulans* varieties (Table 5). In salty environments, the Na^+^ gradient possibly represents an alternative energy source for the influx of P_i_[54].

The most halotolerant of all of the species that have been analysed, *H. werneckii*, contains the highest number of transporter genes (30 genes), although it should be noted that the number is doubled due to the recent whole-genome duplication of *H. werneckii*[54]. The second largest number of transporter genes is present in *C. heterostrophus* (19 genes), which is not a halotolerant species (although, of note, it has not been studied in this respect), and is not found in hypersaline habitats. The high number of plasma-membrane K^+^ transporters of *A. pullulans* (seven or eight transporters across the four varieties) is in even starker contrast with the most halophilic fungus known, the basidiomycete *W. ichthyophaga.* The latter has only one Trk-encoding gene, and has no homologues of the Tok, Hak or Acu transporters [91].

When comparing only these four *A. pullulans* varieties, the most halotolerant *A. pullulans* var. *pullulans* has neither the highest number nor the largest diversity of transporters. Furthermore, although in *S. cerevisiae* the Ena pumps are the major determinant of salt tolerance (for review, see [117]), *A. pullulans* var. *pullulans* even has one Ena gene homologue less than the other *A. pullulans* varieties. Although the number of alkali-metal cation-transporter genes in *A. pullulans* is higher than in many related fungi, this difference is not particularly outstanding. It appears that the diversity and the total number of alkali-metal cation-transporter genes are (at best) only approximate predictors of halotolerance.

Only one gene for the plasma-membrane proton-exporting ATPase Pma is present in each of these four *A. pullulans* varieties. In contrast, *H. werneckii* has four copies of this gene, and their expression is salt responsive [54]. Pma pumps are responsible for establishing the electrochemical gradient of protons across the plasma membrane, and through this, they supply the energy to the secondary active symporters and antiporters [125, 126], among which there are also those responsible for maintaining ion homeostasis under hypersaline conditions.

In addition to the Pma pumps, other transporters might be responsible for the building of the electrochemical gradient of protons. Type 1 microbial rhodopsins can function as proton pumps to establish the electrochemical gradient for ATP production. This process has already been demonstrated in another dothideomycetous fungus, *Leptosphaeria maculans*[127]. An alternative way to build this proton-motive force would not only be useful in hypersaline conditions, as has already been proposed for the extremely halotolerant *H. werneckii*[128], but it might also be of importance in the light-exposed, oligotrophic biology of *A. pullulans*; e.g., to rapidly resume metabolism after cryptobiosis. The search for bacteriorhodopsins revealed at least two gene copies in the genomes of all four of the *A. pullulans* varieties (with three copies in *A. pullulans* var. *melanogenum*). The copies within these genomes differ in terms of their spliceosomal intron numbers and positions. According to our reconstructed phylogeny, these bacteriorhodopsin copies with one and two introns formed monophyletic groups (orthologues; see Additional file 9), and appear to represent old duplications before the split of the lineages within *A. pullulans*. However, *A. pullulans* var. *melanogenum* contains an additional third copy, suggesting either a recent duplication in this *A. pullulans* variety or a loss of a paralogue in the other *A. pullulans* varieties.

Although bacteriorhodopsins from *A. pullulans* have not yet been used for biotechnological purposes, they can be considered for the many different applications that this family of proteins has; e.g., mainly for optical appliances, but also for therapeutic/medical applications and research, including in optogenetics and bioelectronics [129, 130].

### (iii) Description of new *Aureobasidium*species

**Figure 5:**
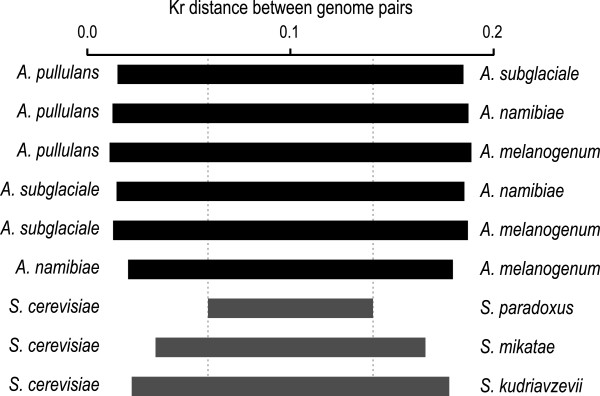
**Kr distances between the genomes of** ***Aureobasidium pullulans*** **and selected pairs of genomes of** ***Saccharomyces cerevisiae, Saccharomyces mikatae*** **, and** ***Saccharomyces kudriavzevii*** **.** Distances are represented by the lengths of the horizontal bars.

The genus *Aureobasidium* (Dothideales, Ascomycota, Dothideomycetes) is a conglomerate of cosmopolitian taxa. The correct taxonomic distinction of the phylogenetic clusters of this genus is of great importance for their industrial applications and investigations of their medical relevance. The analysis of the Kr distances between the genome pairs and the number of amino-acid substitutions per site of the aligned proteomes shows that the differences between these four *A. pullulans* varieties are comparable or larger than those observed between *S. cerevisiae* and three of its relatives, and especially its closest relative, *Saccharomyces paradoxus* (Figure 5, Additional file 10). Substantial differences between the genomes of these four *A. pullulans* varieties were also observed when their scaffolds were aligned with Mummer 3.23 (Additional file 11). Thus, in combination with previous knowledge, this suggested an elevation of these four *A. pullulans* varieties to the species level.

#### Elevation of these four A. pullulans varieties to the species level

A revised concept for the species *A. pullulans* is presented here. These four varieties of *A. pullulans* are elevated to the species level based primarily on the phylogenetic analysis of their whole genome sequences, as well as on their phylogenetically relevant genes.

*Aureobasidium melanogenum* (Hermanides-Nijhof) Zalar, Gostincar, Gunde-Cimerman, stat. nov. MycoBank MB 807698.

Basionym: *Aureobasidium pullulans* var. *melanogenum* Hermanides-Nijhof, Stud. Mycol. 15: 161, 1977. MycoBank MB352628.

*Aureobasidium subglaciale* (Zalar, de Hoog & Gunde-Cimerman) Zalar, Gostincar, Gunde-Cimerman, stat. nov. MycoBank MB 807700.

Basionym: *Aureobasidium pullulans* var. *subglaciale* Zalar, de Hoog & Gunde-Cimerman, Stud. Mycol. 61: 33, 2008. Mycobank MB512380.

*Aureobasidium namibiae* (Zalar, de Hoog & Gunde-Cimerman) Zalar, Gostincar, Gunde-Cimerman, stat. nov. MycoBank MB807701.

Basionym: *Aureobasidium pullulans* var. *namibiae* Zalar, de Hoog & Gunde-Cimerman, Stud. Mycol. 61: 34, 2008. Mycobank MB512381.

For the synonyms, see the previously published taxonomic study of Zalar et al. [19].

#### Taxonomic markers for distinguishing the new Aureobasidium species

Species/varieties within the genus *Aureobasidium* have most often been distinguished based on multilocus analyses of different regions of the rDNA gene clusters. The ITS region has been most commonly used [19, 47–49], although it is recognised as insufficient in certain genera [131]. Based on the ITS rDNA, the distinction of *Aureobasidium pullulans* was possible, but the resolution based on other household genes was better [19]. Therefore, the different household genes used earlier in the phylogenetic analyses of *Aureobasidium* and of other genera were compared here. The selected genes encode for the following proteins: actin (*ACT*), β-tubulin (*BTUB*), calmodulin (*CAL*), chytin synthase (*CHS*), NAD-dependent glycerol-3-phosphate dehydrogenase (*GPD*), mini-chromosome maintenance proteins essential for initiation of eukaryotic genome replication (*MCM7*), RNA polymerase 2, the largest subunit (*RPB1*), RNA polymerase 2, the second-largest subunit (*RPB2*), and translation elongation factor 1α (*TEF1α*). *CAL* and *CHS* are present in several different copies in these four *Aureobasidium* taxa and are thus less appropriate for use in phylogenetic analyses. Other markers were identified as single-copy genes in the genomes.

The overview of these genes – their length, number of exons/introns, possible candidate regions for phylogenetic markers in *Aureobasidium* species, and markers/regions already used in the phylogenetic analyses of *Aureobasidium* species are provided in Table 6.

**Table 6:** **Household genes established as fungal phylogenetic markers**

Gene	Length (bp)	Number of exons/introns	Region used as marker/length/number of variable positions (vp) in ***Aureobasidium*** species	Previous use in ***Aureobasidium*** taxonomy/phylogeny (analysed region and reference)
*ACT*	1587–1605	6/6	Exon 5/722 bp/39 vp	-
*GDP*	1378–1462	3/2	Exon 2/1115 bp/272 vp	-
*MCM7*	2456–2462	3/2	Exon 2/2285 bp/562 vp	-
*BTB*	1634–1645	6/6	Exon 3/exon 5/700 bp/103 vp	Exon 3 to partial exon 5 (450 bp) [19, 49, 132]
*TEF1A*	1799–2006	6/5	Exon 3/1139 bp/87 vp	Intron 3 (260 bp) [19]; exon 3 (1000 bp) [49]
*RPB1*	5442–5461	4/3	Exon 3/4898 bp/1123 vp	Exon 2 to partial exon 3 (644 bp) [131]
*RPB2*	3820–3831	2/1	Exon 1/3608/803 vp	Exon 1 (3500 bp) [133, 134]; part of exon 1 (755–1100 bp) [49]

Properties of the *Aureobasidium* species homologues of phylogenetic markers that can be used to distinguish *Aureobasidium* species originating from this study.

The sequences of *ACT*, *GPD* and *MCM7* have not yet been used in phylogenetic studies of *Aureobasidium*, although they represent appropriate candidate regions for such studies [135]. The positions of the introns in these genes are conserved in all four of the investigated genomes. To evaluate the usefulness of the listed gene exons and introns as barcodes for species identification or as phylogenetic marker loci, further analyses based on a larger set of *Aureobasidium* strains are required.

Comparisons of these loci show substantial differences among the sequenced *Aureobasidium* species. The largest variation is observed in the *GPD* gene. The lowest pairwise distance is between *A. melanogenum* and *A. namibiae* (0.158), and the highest between *A. subglaciale* and *A. melanogenum* (0.214). The smallest variation among these studied genes is in the *ACT* gene, where the pairwise distances range from 0.071 in *A. melanogenum versus A. namibiae*, to 0.092 in *A. pullulans versus A. melanogenum* (Table 7).

**Table 7:** **Pairwise distances for the set of single-copy household genes in the four** ***Aureobasidium*** **species**

Species 1	Species 2	Pairwise distances of genes						
		ACT	BTUB	RPB1	RPB2	MCM7	GPD	TEF
*A. pullulans*	*A. subglaciale*	0.082	0.092	0.156	0.156	0.186	0.161	0.092
*A. pullulans*	*A. melanogenum*	0.092	0.098	0.165	0.157	0.164	0.201	0.075
*A. subglaciale*	*A. melanogenum*	0.089	0.107	0.164	0.140	0.205	0.214	0.093
*A. pullulans*	*A. namibiae*	0.084	0.092	0.167	0.148	0.188	0.185	0.067
*A. subglaciale*	*A. namibiae*	0.088	0.100	0.155	0.144	0.205	0.195	0.094
*A. melanogenum*	*A. namibiae*	0.071	0.078	0.134	0.107	0.156	0.158	0.049

The genome sequences generated here also allowed the correct identification of two other ‘*A. pullulans*’ strains for which the genomes had been sequenced in the past and are publicly available. Strain ATCC 62921 (http://genome.fungalgenomics.ca/), which is of an unknown origin, but it has been used in numerous industrial applications, is confirmed as *A. pullulans*. The sequenced strain AY4, which was isolated from a human patient [36], is identified as *A. melanogenum* according to the above-presented species concept. The new species *A. melanogenum* also includes all other infection-causing ‘*A. pullulans*’ strains. Of the other commonly used ‘*A. pullulans*’ strains, strain NRRL Y-6220 is one of the commonly used strains for pullulan production, and it is related to *A. melanogenum*. On the other hand, the strain R106, which is used for aureobasidin A production, could not be reliably classified with the publicly available sequences, since in the phylogenetic analyses it was placed in a lineage separate from the here-described four species. The two *Aureobasidium* strains that are sold as commercial agricultural biocontrol products (DSM14940, DSM14941) remain in the species *A. pullulans* – for this purpose it is important to note that the species *A. pullulans* does not contain any potentially opportunistic pathogens, as these are placed in the newly described *A. melanogenum*.

#### Homothallic MAT loci and evidence for sexuality

A possible teleomorph of *A. pullulans*, *Columnosphaeria fagi* (H.J. Huds.) M.E. Barr, was suggested on the basis of the ITS sequence similarity [136], although this was not further confirmed either by a multilocus analysis or by a single-spore re-isolation of the fungus. Due to the lack of the recombination between the four previous *A. pullulans* varieties, it was concluded that *A. pullulans* is strictly clonal, although recombination within the four phylogenetic clusters of *A. pullulans* strains (at that time recognised as varieties) was not definitively ruled out [19]. However, the redefinition of these varieties as the four *Aureobasidium* species (see above) implies that the lack of the recombination between the varieties might be simply a consequence of interspecific reproduction barriers and not a sign of asexuality. The genome sequences of the four reclassified *Aureobasidium* species reveal additional details about their possible mating strategies.

The sexual reproduction of ascomycetous fungi is orchestrated by the presence of different arrangements of mating-type genes that encode the key transcription factors at one or more of the MAT loci: the *MAT1-1-1* gene encodes a protein with an alpha1 domain, and the *MAT1-2-1* gene encodes a protein with a high mobility group (HMG) box [137].

**Figure 6:**
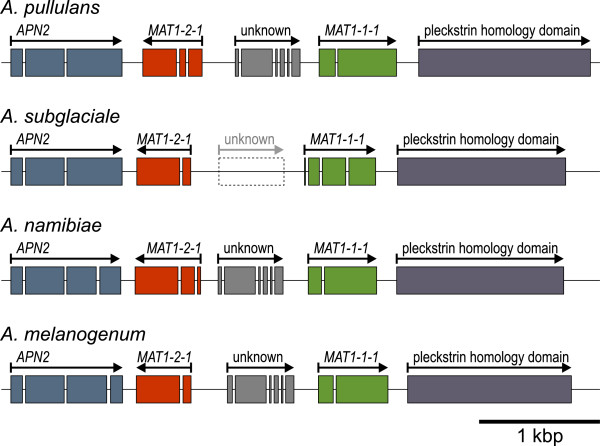
**Configuration of the homothallic** ***Aureobasidium*** **species MAT loci and adjacent genes.** Names of the four new *Aureobasidium* species are indicated above the gene models. Black boxes, exons; arrows, direction of transcription of the gene.

One mating type locus can be clearly recognised in each of the four investigated *Aureobasidium* species. The mating-type locus in all four of these species has a conserved organisation, and contains one of each of the *MAT1-1-1* and *MAT1-2-1* homologues in opposite orientations (Figure 6). This structure of the homothallic MAT locus, where two *MAT* idiomorphs are linked but not fused on the same locus, is also present in some other Dothideomycetes (e.g. *Cochliobolus* species, *Stemphylium* species) [137, 138]. Another set of genes in a syntenic region of all four of these *Aureobasidium* species (Additional file 12) might have the role of the second mating type locus, *MAT2*.

There is an open reading frame of an unknown function between the *MAT1-1-1* and *MAT1-2-1* genes in three of the studied genomes, with the genome of *A. subglaciale* being the exception. In *A. subglaciale*, this open reading frame is still present as a pseudogene, but due to a substantial degeneration, its structure can be discerned only approximately. In Sordariomycetes and Eurotiomycetes, the MAT locus frequently lies between the homologues of the *S. cerevisiae APN2* and *SLA2* genes [54, 137, 139]. In the here investigated *Aureobasidium* species, this is the case for the *APN2* homologues (Figure 6); while the homologues of *SLA2* are located on the same scaffold, they are separated from the *MAT* genes by a substantial distance (70 to 500 kbp).

The single-copy homologues of other genes involved in mating were found outside of the mating loci, including G-protein-coupled receptor for alpha-factor (Ste2 in *S. cerevisiae*) and a-factor (Ste3), and the transcription factor that activates genes involved in mating or pseudohyphal/ invasive growth pathways (Ste12). Out of nine meiosis-specific genes listed by Malik et al. [140], five were identified (homologues of *S. cerevisiae SPO11*, *MSH4*, *MSH5*, *MER3, REC8*). Interestingly, the homologue of *MER3*, a DNA helicase that is involved in crossing over [141], is present in three copies in *A. melanogenum*, and in two copies in *A. pullulans*, *A. subglaciale* and *A. namibiae*.

The roles of absent genes might be provided by alternative proteins. For example, the species *A. pullulans* contains two homologues of Rad51, which is a protein involved in the repair of double-strand DNA breaks. This might replace Dmc1, which performs the same function, but only in meiosis. Furthermore, the absence of the *DMC1* homologue in sexual fungi is not unusual [142].

According to their genetic design, all four of the *Aureobasidium* species can be considered as homothallic (self-compatible, or self-fertile), which implies that a single strain is capable of sexual reproduction even in an axenic culture. However, the existence of mating genes is not conclusive evidence for sexual recombination, as these genes might also have other roles, as has been reported in other species of fungi [143]. As a teleomorph of the previously recognised *A. pullulans* has not been described yet, the ability of these four *Aureobasidium* species to undergo sexual reproduction needs to be confirmed experimentally.

## Conclusions

*Aureobasidium pullulans*, as it was previously defined, was considered a species of exceptional adaptability. This was reflected in its great phenotypic plasticity, polyextremotolerance, and the ability to survive across a wide variety of habitats. A part of this diversity can be explained by the relatively wide definition of this species. However, with the accumulation of new knowledge, it had become clear that smaller, well-defined groups of strains can be recognised within *A. pullulans*. These were initially described as varieties [19], but this comparison of their genomic data shows that the differences between these varieties are large enough to justify their redefinition as four separate *Aureobasidium* species: *A. pullulans*, *A. melanogenum*, *A. subglaciale* and *A. namibiae*.

Even single strains of *A. pullulans* can show wide adaptability, and this is reflected in the great diversity of several groups of genes. This diversity can reach, or even surpass, the gene diversity of more specialised related species, even though almost all of them have larger genomes. The investigated genomes of these four *Aureobasidium* species contain an abundance of different families of extracellular enzymes, which are important for the degradation of the plant cell-wall material. Their diversity is comparable to the phylogenetically related plant pathogens. The differences in the abundance of the extracellular enzymes and also in the higher numbers of sugar transporters reflect the ecological preferences of the four studied *Aureobasidium* species, especially for the plant-associated lifestyle of *A. pullulans.* Furthermore, for example, all four of the *Aureobasidium* genomes presented here have genes for the alternative K^+^ importers Hak and Acu, which are not found even in the extremely halotolerant *H. werneckii* or in the halophilic *W. ichthyophaga*. Also, when considering the mating strategy, these four *Aureobasidium* species appear to be more flexible than the specialised *H. werneckii* and *W. ichthyophaga*: the four *Aureobasidium* genomes all contain a homothallic mating type locus, whereas *H. werneckii* is heterothallic, and *W. ichthyophaga* lacks the mating genes altogether.

The genome sequences of the described four *Aureobasidium* species are expected to facilitate the exploitation of the substantial biotechnological potential of these fungi. For most of the interesting genes, at least four different genome copies are now available for study, with potentially different traits of the proteins that they encode. Additionally, we propose the elimination of the decade-old annotation error in the putative pullulan synthase gene. Furthermore, the genes for the degradation of a variety of compounds from plant polysaccharides to plastics and aromatic compounds should lead to more efficient industrial use of these *Aureobasidium* species, as well as help in the selection or construction of the best strains for any given purpose.

The polyextremotolerant character of the investigated *Aureobasidium* species indicates that they have efficient stress-combating mechanisms, some of which have been described here. These mechanisms can potentially be used in the future to improve the stress tolerance of economically important microorganisms or plants. Last but not least, as the opportunistic human pathogens belong only to *A. melanogenum*, the phylogenetic redefinition of the previously recognised *A. pullulans* will facilitate the identification of the more problematic strains and help in limiting their use in applications in which they might represent potential health risks for workers or consumers.

## Methods

### Strains, growth conditions and microscopy

*Aureobasidium pullulans* var. *pullulans* (EXF-150, CBS 100280) was isolated from the hypersaline waters of the Sečovlje solar saltern (Slovenia). *Aureobasidium pullulans* var. *melanogenum* (EXF-3378, CBS 110374) was isolated from a public fountain in Bangkok (Thailand). *Aureobasidium pullulans* var. *subglaciale* (type strain; EXF-2481, CBS 123387) was isolated from the subglacial ice of the Kongsvegen glacier on Spitsbergen (Svalbard, Norway). *Aureobasidium pullulans* var. *namibiae* (type strain; EXF-3398, CBS 147.97) was isolated from dolomitic marble in the Namib Desert (Namibia). All of these strains are preserved in the Ex Culture Collection of the Department of Biology, Biotechnical Faculty, University of Ljubljana (Infrastructural Centre Mycosmo, MRIC UL) and at the Centraalbureau voor Schimmelcultures (CBS, Utrecht, The Netherlands).

These cultures were grown at 28°C in a rotary shaker (180 rpm) in the defined yeast nitrogen base medium (YNB) of 0.17% (w/v) yeast nitrogen base, 0.08% (w/v) complete supplement mixture (both Qbiogene), 0.5% (w/v) ammonium sulphate, and 2% (w/v) glucose, in deionised water. The pH was adjusted to 7.0 prior to autoclaving. For the isolation of RNA for transcriptome sequencing of *A. pullulans* var. *pullulans* and *A. pullulans* var. *subglaciale*, which was used for gene annotation, a variety of conditions was used: (1) YNB; (2) YNB with additional 10% (w/v) NaCl; (3) YNB with 2% (w/v) raffinose or (4) 31.3% (w/v) glycerol instead of glucose; (5) YNB with 49% (w/v) sorbitol; (6) yeast peptone glucose medium (1% [w/v] yeast extract, 2% [w/v] peptone, and 2% [w/v] glucose, in deionised water); (7) minimal medium (2% [w/v] glucose, macroelements [w/v]: 0.6% NaNO_3_, 0.15% KH_2_PO_4_, 0.05% MgSO_4_ × 7H_2_O, 0.05% KCl, microelements [w/v]: 0.01% EDTA, 0.0044% ZnSO_4_, 0.001% MnCl_2_ × 4H_2_O, 0.00032% CoCl_2_ × 6H_2_O, 0.00032% CuSO_4_ × 5H_2_O, 0.00022% (NH_4_)_6_Mo_7_O_24_ × 4H_2_O, 0.00147% CaCl_2_ × 2H_2_O, 0.001% FeSO_4_ × 7H_2_O, in deionised water); (8) minimal medium with 2% (w/v) cellulose instead of glucose; (9) YNB and an incubation temperature of 12°C; (10) YNB and incubation until mid-exponential phase followed by heat shock at 42°C for 45 min; (11) YNB and incubation until stationary phase; (12) YNB and incubation until mid-exponential phase, followed by addition of 0.32 mM H_2_O_2_ for 5 min; and (13) YNB and incubation until mid-exponential phase, followed by centrifugation and further resuspension either in YNB at pH 2 and a 4-h incubation (one generation), or (14) in Tris base at pH 10 for 30 min. Unless otherwise noted, the cells were harvested in the mid-exponential growth phase, using a 10-min centrifugation at 5000× *g*, and immediately frozen in liquid nitrogen.

For photography of the macromorphology, the four *A. pullulans* varieties were grown on malt extract agar (MEA) according to Blakeslee, containing 2% malt extract, 0.1% peptone, 2% glucose and 2% agar (all Merck), for 30 days at 24°C.

For microscopic images, slide cultures were prepared on MEA blocks in moist chambers, and they were incubated for 7 days at 25°C in the dark. The cover slips were carefully removed and mounted in 60% lactic acid for microscopic observation under the Olympus BX51 microscope, using an Olympus DP12 camera, and the DP software.

### DNA and RNA isolation

Cells frozen in liquid nitrogen were homogenised using a pestle and a mortar. Their DNA was isolated using the phenol/ chloroform/ isoamyl alcohol method, modified for DNA isolation from filamentous fungi, as described previously [144]. The quality and quantity of the DNA was evaluated spectrophotometrically with NanoDrop 2000 (Thermo Fisher Scientific, USA), and on standard 1% agarose gel electrophoresis, with the molecular weight also checked.

The RNA was isolated using TRI Reagent (Sigma-Aldrich, Germany), according to the manufacturer instructions. Possible DNA contamination was removed with DNAse I (Thermo Fisher Scientific – Fermentas, Lithuania), and the integrity and purity of the RNA was evaluated spectrophotometrically with NanoDrop 2000 (Thermo Fisher Scientific, USA) and by capillary electrophoresis (Agilent 2100 Bioanalyser; Agilent Technologies, USA).

### Genome sequencing and assembly

The draft genome of *A. pullulans var. pullulans* was sequenced with Illumina HiSeq using two libraries, fragment, 270-bp-insert size (2× 150 bp reads) and CLIP, 4-kbp-insert size (2× 100 bp reads). Each fastq file was QC filtered for artifacts/ process contamination, and subsequently assembled together with AllPathsLG version R38445 [145], to produce a 172× coverage 29.6 Mbp assembly in 186 scaffolds (L50 = 1.2 Mbp) and 209 contigs (L50 = 779.8 kbp). The 37121 bp mitochondrion genome was assembled separately in a single contig with AllPathsLG.

The genomes of *A. pullulans* var. *namibiae*, *A. pullulans* var. *melanogenum*, and *A. pullulans* var. *subglaciale* were sequenced using a single fragment Illumina library with 270-bp-insert size (2× 150 bp reads). After filtering for artifacts/ process contamination, the sequence data were assembled with Velvet 1.2.03 [146]. The resulting assembly was used to create a long mate-pair library with an insert of 3000 +/-300 bp, which was then assembled together with the original Illumina library with AllPathsLG release version R42328 [145]. The assembly statistics are summarised in Table 1.

### Transcriptome sequencing and assembly

mRNA was purified from total RNA using Absolutely mRNA™ purification kits (Stratagene) and Dynabeads® mRNA purification kits (Invitrogen), and chemically fragmented to 200 bp to 250 bp (Ambion). The mRNA was reverse transcribed with SuperScript II, using random hexamers. Second-strand cDNA was synthesised using a dNTP/dUTP mix (Thermo Scientific), *E. coli* DNA ligase, *E. coli* DNA polymerase I, and *E coli* RnaseH (Invitrogen). The fragmented cDNA was treated with end-pair, A-tailing, and adapter ligation, using TruSeq Sample Preparation kits (Illumina) and KAPA-Illumina library creation kits (KAPA biosystems). Second-strand cDNA was removed by AmpErase UNG (Applied Biosystems), to generate strandedness. qPCR was used to determine the concentrations of the unamplified libraries. The libraries were sequenced on Illumina Hiseq.

In all, 120 million paired-end 100-bp Illumina HiSeq 2000 reads of stranded RNA-seq data were used as input for the *de-novo* assembly of *A. pullulans* var. *subglaciale* EXF-2481. One-hundred and sixty-four million paired-end 150-bp Illumina HiSeq 2000 reads of stranded RNA-seq data were used as input for the *de-novo* assembly of *A. pullulans* var. *pullulans* EXF-150. The reads were assembled into consensus sequences using Rnnotator 2.4.14 [147], which takes short read sequences as input, and outputs assembled transcript contigs. The reads were trimmed and filtered for quality, low-complexity, adapter and duplicates, and assembled with Velvet 1.2.03 [146]. The minimum contig length was set at 100 bp. The read depth minimum was set to 3 reads. Redundant contigs were removed using Vmatch 2.1.3, and contigs with significant overlap were further assembled using Minimus2, with a minimum overlap of 40 bp. Contig post-processing included splitting misassembled contigs, and contig extension and polishing using the strand information of the reads. Single base errors were corrected by aligning the reads back to each contig with BWA, to generate a consensus nucleotide sequence. Post-processed contigs were clustered into loci and putative transcript precursors were identified. The accuracy, completeness and contiguity of the assembled contigs were checked using Blat alignment [148] of the contigs to the available genomic reference. Here, 95.87% of the EXF-2481 contigs mapped to the genome, and 90.24% of the EXF-150 contigs mapped to the genome (threshold ≥95% of the contigs).

### Genome annotation

All four of these genome assemblies of *A. pullulans* were annotated using the Pipeline JGI annotation [149], which combines several gene prediction and annotation methods, and integrates the annotated genome into the web-based fungal resource MycoCosm [150], for comparative genomics.

Before gene prediction, assembly scaffolds were masked using RepeatMasker [151] and RepBase library [152], with the most frequent (>150 times) repeats recognised by RepeatScout [153]. The following combination of gene predictors was run on the masked assembly: *ab-initio* Fgenesh [154] and GeneMark [155], trained for specific genomes; homology-based Fgenesh + [154] and Genewise [156], seeded by BLASTx alignments [157] against the NCBI-NR protein database; and transcriptome-based CombEST (Zhou *et al.*, personal communication). In addition to protein-coding genes, tRNAs were predicted using tRNAscan-SE [158]. All of the predicted proteins were functionally annotated using SignalP [159] for signal sequences, TMHMM [160] for transmembrane domains, InterProScan [161] for the integrated collection of functional and structured protein domains, and protein alignments to NCBI-NR, SwissProt (http://www.expasy.org/sprot/), KEGG [162] for metabolic pathways, and KOG [163] for eukaryotic clusters of orthologues. Interpro and SwissProt hits were also used to map the gene-ontology terms [164]. For each genomic locus, the best representative gene model was selected based on a combination of protein similarity and EST support.

### Shared, unique and duplicated genes

The numbers of shared and unique proteins of all four of the *A. pullulans* varieties were determined using all-against-all blastp (included in blast 2.2.25+) of their whole proteomes, with the e-value cut-off at 10^-10^[157]. Additionally, multigene families were predicted with the MCL, to cluster the proteins based on sequence similarity, using blastp alignment scores between proteins as a similarity metric [165]. The numbers of unique and shared clusters were retrieved from MycoCosm [166].

### Mating loci, secreted proteins, membrane transporters

Mating loci were identified by using the JGI online blastp programme (e-value cut-off, 10^-5^). The *S. cerevisiae* Mat1-1-1 and Mat1-2-1 proteins were used as sequence queries. Additionally, other proteins characteristic of mating type loci were identified for each of the *A. pullulans* varieties, as described in [91]. A blastp search was performed for pheromone precursors (α and α-factors), MAPK scaffold (Ste5), transcription factors (Ste12) and pheromone factor receptors (Ste2, Ste3) [167], and also for the representative set of homologues of meiotic genes from *S. cerevisiae* (i.e., a ‘meiosis detection toolkit’) [140].

The prediction of secreted proteins was performed in several steps. First SignalP [168] was used to search for signal peptides in all of the predicted proteins of each of the four *A. pullulans* varieties, with the default cut-off D-value of 0.43. Proteins that were predicted by TargetP [169] to localise in other cellular compartments, as well as those containing predicted transmembrane regions by TMHMM [170], were removed from the selection. This produced a list of proteins that was expanded with the help of the multigene families predicted with the Markov clustering algorithm (MCL), as described below. Clusters that contained at least half of the proteins predicted as secreted (above) were automatically included in the list of secreted proteins (2506 proteins). This covered the cases where homologues of one or two of the *A. pullulans* varieties were not included in the original list due to annotation errors, or for other reasons. Clusters that contained less than half of the predicted secreted proteins (2274 proteins) were further analysed with online predictors CELLO [171] and WegoLoc [172], the latter with fungal BaCelLo and Höglund databases, with an e-value threshold of 1 × e^-10^ and a multiplex threshold of 1. The proteins were considered as secreted if they were predicted as such by at least three of four methods (our method described above, and three online predictors). The functions of predicted secreted proteins were assigned manually with the help of the available data about each of these proteins: placement into multigene families, gene ontology terms, presence of PFAM domains, functions of similar proteins from the NCBI-NR protein database, and results of CAZymes Analysis Toolkit [173] and MEROPS [72].

The analysis of the major facilitator transporters included all of the *A. pullulans* predicted protein sequences containing the PFAM domain (PF00083), which is characteristic of sugar transport proteins. The proteins were classified into sugar-specific families based on the phylogenetic analyses (performed as described below), which included the related proteins from other species. These were identified with blastp against the NCBI-NR protein database [157], or were retrieved from the Transporter Classification Database (http://www.tcdb.org/superfamily.php, 1. 8. 2013), and the MFS family sugar transporters of *S. cerevisiae* S288C were retrieved from the TransportDB database (http://www.membranetransport.org/, 1. 8. 2013).

Genes for alkali-metal cation transporters from *Mycosphaerella graminicola*, *S. cerevisiae* and *C. neoformans* were recovered from public databases, as described in [54], while the genes from *H. werneckii* were published in [54]. Homologues from other Dothideomycetes with sequenced genomes (the same as those used for phylogenetic analyses; see below) were recovered from the Genome Portal of the JGI, and were considered homologous to the analysed proteins from *A. pullulans* if they formed the same MCL cluster [166]. A second set of trees (for comparison purposes; not shown) was generated by applying a maximum parsimony method as implemented in the Mega software, version 5.05 [174]. The specificity of P-type pumps was inferred by phylogenetic analysis of all of the P-type cation transporters found from *A. pullulans* and their homologues with known function from other fungi, as described in [54].

Melanin polyketide synthase genes were identified by searching for a homologue of *ALM1* from *Alternaria alternata* [GenBank:AB665444]. Homologues from *Magnaporthe grisea* [GenBank:L22309, GenBank:AY846877] were used as queries in the search for the trihydroxynaphthalene reductase-like gene and tetrahydroxynaphthalene reductase-like genes. Protein sequences of orthodox aquaporins Aqy1p and Aqy2p, and of aquaglyceroporins Fps1p and Yfl054p, from *S. cerevisiae* (sequences from the *Saccharomyces* genome database; http://www.yeastgenome.org/), were used as queries for the aquaporin genes. Other genes discussed were found with the help of blastp and MCL clustering data on the Genome Portal of the JGI [166], and analysed with various search strategies against the NCBI-NR protein database and the *Saccharomyces* Genome database.

### Phylogenetic analyses

For the analysis of phylogenetic relationships of the four *A. pullulans* varieties and related species, a super alignment of the selected fungal proteomes was constructed with the Hal pipeline [175], allowing for no missing data. In addition to all four of the *A. pullulans* varieties, several other proteomes available on the Genome Portal of the Department of Energy Joint Genome Institute [166] were included: *Baudoinia compniacensis*[51], *C. fulvum*[176], *C. heterostrophus*[51], *Hysterium pulicare*[51], *M. graminicola*[177], *N. crassa*[82], *Sclerotinia sclerotiorum*[178], and *Tuber melanosporum*[179]. *A. nidulans*[180] was used as an outgroup. Hal produced a conservative alignment of 812692 amino acids. Poorly aligned positions and positions with gaps were removed with Gblocks 0.91b [181]. Stringent parameters were used: the maximum number of contiguous non-conserved positions was limited to 5 amino acids, and the minimum length of a block to 15 amino acids. This produced a 373159-bp-long alignment, which was used for the estimation of phylogeny.

The best protein evolution model was estimated with ProtTest 3.2.1 [182]. The species tree was generated with the PhyML 3.1 software [183]. Approximate Bayes (aBayes) branch supports were calculated. The analysis was run using the LG model of evolution. The ProtTest estimate of the α-parameter of the γ-distribution of six substitution rate categories (0.791), and the determined proportion of invariable sites (0.305) were used. The tree was then calibrated with the r8s software [184], by assigning the root of the tree to an arbitrary value of 1.

Gene phylogenies (alkali-metal cation transporters, major facilitator superfamily transporters, bacteriorhodopsins, components of HOG, aquaporins, scytalone dehydratases) were estimated by aligning the protein sequences with the MAFFT software in the ‘--auto’ mode [185]. The tree was generated with the PhyML 3.1 software [183], as described above, by using the model of protein evolution, the α-parameter, and the proportion of invariable sites, as estimated by ProtTest 3.2.1 [182].

### Genome alignment and genome distances

The whole genome alignments between all of the possible pairs of these four genomes were calculated with the promer algorithm, as implemented in Mummer 3.23, and plotted with the mummerplot utility [186]. All of the parameters were the same as described by Hane et al. [187], except that instead of discarding scaffolds <500 kbp, only scaffolds <200 kbp were discarded.

The distances between these four *A. pullulans* varieties and between four *Saccharomyces* species (for comparison purposes) were estimated by using two methods. First, the *K*_*r*_ distances were calculated with the genomediff software included in the GenomeTools library [188]. This alignment-free calculation produces Jukes-Cantor corrected divergences between the pairs of genomes as output. As a second approach, the proteomes were aligned with the Hal pipeline [175]. The conservative alignment was used for the estimation of the number of amino-acid substitutions per site, in Mega software version 5.05 [174]. The Poisson correction model was used, and all of the positions containing gaps or missing data were removed prior to analysis, which resulted in a 698915-amino-acid-long alignment.

### CAFE analyses

Analysis of protein family expansions and contractions was performed with the CAFE 3.0 software [71]. Pfam domains of selected fungal proteomes were identified with a stand-alone Pfam scanner and a database downloaded on 30.1.2013 [189]. Additionally, multigene families predicted with the MCL, as described above, were used as a second variant of grouping the predicted proteins. This was used to produce a Table with the numbers of proteins belonging to specific protein families in each of the analysed species. The Table was used as the input of CAFE, together with the chronogram constructed from the whole proteomes, as described above.

This produced a list of groups of proteins with significant expansions/contractions. This was manually checked, and the groups with potential roles in stress tolerance or biotechnological use were selected for further analyses.

### antiSMASH analysis

The antiSMASH 2.0 software [190] was used for the identification of the secondary metabolite biosynthesis clusters in the genome sequences. antiSMASH is the first comprehensive pipeline that can identify biosynthetic loci that cover the whole range of known secondary metabolite compound classes (polyketides, non-ribosomal peptides, terpenes, aminoglycosides, aminocoumarins, indolocarbazoles, lantibiotics, bacteriocins, nucleosides, β-lactams, butyrolactones, siderophores, melanins, and others) [66]. Nucleotide sequences of all four of the *A. pullulans* varieties were uploaded to the antiSMASH online site (http://antismash.secondarymetabolites.org/) in FASTA format, and the parameters were set to default, with the additional setting for the DNA of eukaryotic origin.

### Data access

The genome assemblies and annotations can be interactively accessed through the JGI fungal genome portal MycoCosm [150] at http://genome.jgi-psf.org/Aurpu_var_pul1/Aurpu_var_pul1.home.html, http://genome.jgi-psf.org/Aurpu_var_sub1/Aurpu_var_sub1.home.html, http://genome.jgi-psf.org/Aurpu_var_nam1/Aurpu_var_nam1.home.html, and http://genome.jgi-psf.org/Aurpu_var_mel1/Aurpu_var_mel1.home.html, and have also been deposited with DDBJ/EMBL/GenBank under accession [GenBank:AYEO00000000, GenBank:AYYB00000000, GenBank:AYEM00000000, GenBank:AYEN00000000], for *A. pullulans*, *A. subglaciale*, *A. namibiae*, *A. melanogenum*, respectively.

## Electronic supplementary material

Additional file 1: **Alignment of putative pullulan synthetases.** Homologues from the sequenced genomes of the four *A. pullulans* varieties and a previously published gene ([GenBank:AF470619] [57]). ApP, *A. pullulans* var. *pullulans*; ApS, *A. pullulans* var. *subglaciale*; ApN, *A. pullulans* var*. namibiae;* ApM*, A. pullulans* var*. melanogenum.* JGI protein IDs are included. Red, predicted exones; green, introns; blue (arrow), one-nucleotide frameshift insertion. (PDF 13 KB)

Additional file 2: **Secondary metabolite biosynthetic clusters of the four** ***Aureobasidium pullulans*** **varieties.** (XLSX 23 KB)

Additional file 3: **Numbers of carbohydrate-active enzymes in the predicted secretomes of the four** ***Aureobasidium pullulans*** **varieties.** (XLSX 12 KB)

Additional file 4: **Different CAZy glucoside hydrolase families that are involved in degradation of plant cell walls in different fungal genomes.** Blue bars, degradation of hemicellulose (GH10, GH11, GH27, GH29, GH35, GH36, GH39, GH43, GH51, GH53, GH54, GH62, GH67, GH93, GH115); red bars, degradation of cellulose (GH6, GH7, GH12, GH45, GH74, GH94, AA9 (previously GH61)); dark yellow vertical line (left), adapted from [51]; green vertical line (left), adapted from [70]. (PDF 37 KB)

Additional file 5: **Protein tree of scytalone dehydratases.** GenBank accession numbers of individual proteins are listed in the tree. Different gene clusters are marked with different colours. Scytalone dehydratases of the *A. pullulans* varieties are marked in red. (PDF 38 KB)

Additional file 6: **Protein tree of the aquaporins.** The GenBank accession numbers of the individual proteins are listed in the tree. Different gene clusters are marked with different colours, which correspond to previously recognised phylogenetic groups [116]. Aquaporins of *A. pullulans* varieties are marked in red. (PDF 55 KB)

Additional file 7s: **Protein trees of various membrane transporters of Na** ^**+**^ **and K** ^**+**^ **.** Protein trees marked with the names of the *S. cerevisiae* homologues (except for Acu and Hak K^+^ transporters, which are not found in *S. cerevisiae*)*.* The trees (except for Acu and Hak) were rooted with homologous proteins from *C. neoformans* (Trk: [GenBank:XP_570017] and [GenBank:XP_569339]; Tok: [GenBank:XP_568987] and [GenBank:XP_568988]; Nha: [GenBank:XP_569560]; Ena: [GenBank:XP_572412], [GenBank:XP_568029] and [GenBank:XP_570160]; Pho: [GenBank:XP_568082]; Nhx: [GenBank:XP_570596]; Kha: [GenBank:XP_571501]; Vnx: [GenBank:XP_569752]; Mrs: [GenBank:XP_569566]; Pma: [GenBank:XP_568571]) and the root location is marked with an arrow. In addition to genes from *A. pullulans*, homologues from the following fungi were used: *H. werneckii* (Hw), *M. graminicola* (Mg) and *S. cerevisiae* (Sc). For Acu, homologues from *Ustilago maydis* (Um) and *Millerozyma farinosa* (Mf; Pichia farinosa) were used; for Hak, homologues from *N. crassa* (Nc) and *C. albicans* (Ca) were used. The GenBank accession numbers of the individual proteins are listed in the trees. Putative gene duplications leading to the present diversity of these genes in *A. pullulans* are marked with double arrows. ApP*, A. pullulans* var*. pullulans;* ApS*, A. pullulans* var*. subglaciale;* ApN*, A. pullulans* var*. namibiae;* ApM*, A. pullulans* var*. melanogenum.* (PDF 35 KB)

Additional file 8: **Alkali-metal cation transporters of the four** ***Aureobasidium pullulans*** **varieties and related fungi, divided into subgroups as determined by the phylogenetic analysis (Figure** 4 **).** (XLSX 11 KB)

Additional file 9: **Phylogenetic analysis of bacteriorhodopsins from the four** ***Aureobasidium pullulans*** **varieties and related fungi.** Numbers before species names are protein ID numbers of the Joint Genome Institute Genome Portal. The structures of the genes are represented by horizontal bars (exons) and spaces between them (introns). Lengths of the bars are proportional to the lengths of the feature represented. (PDF 22 KB)

Additional file 10: **Distances between the four sequenced genomes of** ***Aureobasidium*** **species and selected** ***Saccharomyces*** **species.** (XLSX 12 KB)

Additional file 11: **Dot-plot comparison of** ***Aureobasidium*** **species scaffolds longer than 200 kbp.** Six-frame translations of scaffolds aligned with Mummer 3.23. Homologous regions are plotted as dots. Scaffolds of each species are displayed ordered by decreasing size along the X and Y axes. Diagonal lines of dots in individual boxes represent syntenic regions. (PDF 7 MB)

Additional file 12: **Putative second mating type locus, MAT2-2.** Putative MAT2-2-1 genes with clearly recognisable HMG box domains (PF00505). Downstream of the putative MAT2-2-1 there is a gene with the Mob1/phocein family (Mob1 kin. reg.; PF03637), and taurine catabolism dioxygenase (TauD; PF02668), and a gene with the major facilitator superfamily (*MFS_1*; PF07690) domain. Upstream of the putative MAT2-2-1 there are two genes, the first encoding a hypothetical protein most similar to GDSL lipase (with no Pfam domain), and the second encoding SNF-2 helicase (PF00176). (PDF 16 KB)

## Abbreviations

antiSMASH: Antibiotics and secondary metabolites analysis shell
CAFE: Computational analysis of gene family evolution
DHN: Dihydroxynaphthalene
GH: Glycoside hydrolases
GT: Glucosyltransferase
HOG: High osmolarity glycerol
MFS: Major facilitator superfamily
MIP: Major intrinsic protein
MAPK: Mitogen-activated protein kinase
NCBI-NR: National center for biotechnology information non-redundant
PL: Polysaccharide lyases.
