# Supplementary material for: Genome sequencing of four Aureobasidium pullulans varieties: biotechnological potential, stress tolerance, and description of new species
Source: BMC Genomics. 2014 Jul 1;15:549. doi: 10.1186/1471-2164-15-549 (PMC4227064; doi:10.1186/1471-2164-15-549)
Supplement: Supplementary file 1 — Additional file 1: Alignment of putative pullulan synthetases. Homologues from the sequenced genomes of the four A. pullulans varieties and a previously published gene ([GenBank:AF470619] [57]). ApP, A. pullulans var. pullulans; ApS, A. pullulans var. subglaciale; ApN, A. pullulans var. namibiae; ApM, A. pullulans var. melanogenum. JGI protein IDs are included. Red, predicted exones; green, introns; blue (arrow), one-nucleotide frameshift insertion. (PDF 13 KB) [file 12864_2014_7061_MOESM1_ESM.pdf]

```
ApP-349889      -----------------AACCA--CAAATTACCT-TTCAAACCACATCCAATTCAAAACT
ApS-3372        CAAACATTCTTGTTTGAAACAACACACTTTTCTGCATCA--CTTCTTCTTGTTGAAAACA
ApN-53761       ----CCTACTTTCTCCTAGACAC--ACCTTTTCATCGCAATAAAA-TCGTTTTCGAATCA
ApM-64747       -----CTACTTCTCCCTAAACACACACATTTTCACTGTGATCAGAGTCTTTCTT-AGACA
AF470619        -----CTACTTCTCCCTAAACA--CACATTTCCACTGTGATCAGATTCTTTTTC-AAACA

ApP-349889      --------------------ATCTCAAGGAATTGTTTC---AACGACAGGA-----TATC
ApS-3372        TCGTCACCGGATACGAATTATTGTCAA-GAGCATACTCAGCATCGAAAACTTTCACATAA
ApN-53761       TCA------A-CACAAGATATACTC----AGCAGTAACACCCAAGTCATCAT--A--CAA
ApM-64747       GCA------AGAACAGAATATCTCC----AGATCTAAT---CTCGAACACCT--ACAACA
AF470619        TCA------GAAACAGGACATCTTC----AGCAGTAAC---CTTGAAAACCT--ACAACA

ApP-349889      ACAATGCATTTCTCCACCACCATTTTCGCCG---CTTTAATCGCCCTCGCAACCGCTTCT
ApS-3372        ATCATGCATTTCTCCATCCTTGCCCTCGCCGCGTCTGTGGCTGCTCTGGCGACTGCATCT
ApN-53761       ACCATGCGCTTCTCCATCCTTGCCTTCGCCGCCTCGCTGGCCACCTACGCAGCTGCATCT
ApM-64747       AACATGCGCTTCTCCATCTTCGCCCTCGCCGCCTCCCTGGCCGCCGTGGCAACTGCTTCT
AF470619        AATATGCACTTCTCCATATTCACCCTCGCCGCCTCCCTGGCCGCCCTGGCAACTGCTTCT

ApP-349889      CCCA-TCATC--------GATCGCGCTCCAACAATCAACACCACACAACCCTTCTACCTC
ApS-3372        CCTACTCGTCGCGTCGCCGGCAAAGTTCCTGTCATCAACAGCACTCAACCCTTCTACCTC
ApN-53761       CCTC-TCATC--------AGCCGCGCACCTACCATCAACGCTACCACGCCCTTCTACCTC
ApM-64747       CCTC-TCGTC--------AAACGCGCACCCATCATCAACAGCACACAACCCTTCTATCTC
AF470619        CCTCTTCGTC--------ACCCGCGCACCCATCATCAACAGCATCAACCCCTTCTACCTC
                    ↑

ApP-349889      CTAACAACCACCACGCCAACCTACACCTCCAACTCCTCCCTCCTGCCAAATGTCTCCCTA
ApS-3372        TTGACCACCGACTCAGAAACATACTCTGACAACGCCACTCTCCTGTCGAACGTATCCTTG
ApN-53761       CTAACAACAAACTCAGCAACCTACTCCAAAGACTCATCTCTGCTCCCCAACGTCTCCTTG
ApM-64747       ATCACAACAAACTCACCAACCTACTCTCAAAACTCTTCATCGCTTCCCAACGTCACTTTG
AF470619        CTCACAACCAACTCACCAACCTACGCTCAAAACTCTTCTTTGCTTGCCAATGTCTCTTTG

ApP-349889      ACCACCCTCTTCGACCCCTACTACCAACCCAACTATCTGTTACGTCTGATCGCCCCCGGC
ApS-3372        ACCACCCTATTCAGCCCTTACTACCAATCAAACTATCTCTTGCGTCTCATTGCTCCAGGT
ApN-53761       ACCACTCTCTTCGACCCTTACTACCAGCCCAACTACCTCCTCCGCCTTATTGCACCTGGC
ApM-64747       ACCACTCTTTTCGACCCCTACTACAAACCTAACTACCTTCTCCGCCTCGCCTATCCCGGC
AF470619        ACCACCCTCTTCGACCCCTACTACCAACCCAACTACCTGCTCCGCCTCACTGGTCCCGGC

ApP-349889      TATGGCTCCGTCCCCCAATTCACCATTTCAAACGGAGTCTTGCACACGCCCGGACAAGGC
ApS-3372        TATGGTAGTGTGCCCCAGTTCACGCTGGGTGACGGTATCCTGCACACTCCTACTAAGGGA
ApN-53761       TATGGCAGTGTGCCTCAATTCACCCTCTCCGATGGCGTTCTGCACACACCTGGCAAAGGA
ApM-64747       TATGGCAGTGTTCCCCAATTTACACTTTCGGACGGAGTGCTGCACTGTCCTGAACAGGGC
AF470619        TATGGTAGTGTTCCTCAATTCACACTTTCGCAGGGAGTGTTGCACTGTCCTGGCCAGGGT

ApP-349889      CCCCATGGCATTGGCAATTATATCTTCAACAGCAGTGACGTGCACACGGGTTCTGAGCTC
ApS-3372        CCTCATGGTATTGGTGACTTCATCTACAACAGCAGCGAAGTTCACACCGGCTCGGAGCTG
ApN-53761       CCCCATGGTATTGGTGACTACATCTACAACAGCACTGAAGTGCACACTGGCTCTGAATTG
ApM-64747       CCCCACGGTATTGGCAACTATATCTTCAACAGCACTGATGTGCATACTGGCTCAGAGTTG
AF470619        CCCCACGGTATCGGCAACTACGTCTTCAGCACCACTGACGTGCACACTGGCTCGGAGTTG

ApP-349889      GAGTTCCGCGCTCAGTTTGAGGGAGAGGGCGATCTGAGTCTTGAACGTGGGTATTTGTTG
ApS-3372        CAGTTCAGGGCTGAGCAAGCAGGACCTGGCGACCTGGGTCTCAAGAACGGCTATCTTCTT
ApN-53761       AATTTCAGGACCCAGTACGAGGGCACTGGAGATCTGAGCCTTGAAAGGGGATACTTGCTG
ApM-64747       CAATTCAGAGCTCAGTACGAGGGAACCGGAGATTTGACTCTCGAGAAAGGATATCTGCTT
AF470619        CAGTTCAGAGCTCAGTATGAGGGCACTGGAGATTTGACTCTCGAAAAGGGATATCTGCTT

ApP-349889      GGTGTCAATGGGTCGAGTGACGGGTGGACGATTTGTGTCGAGGAGTTGGGTCAGAGGGTG
ApS-3372        GGCGTCAACGGAAGCAGCGACGGATGGACAGTATGTGTTGAGGAATTAAGTCAGAGAGTC
ApN-53761       GCTGTCAACGGAAGTACTCATGGTTGGACTATCTGTGTGGAGGAGCTGGGTCAGAGAGTT
ApM-64747       GGTGTGAATGGAAGCACGACTGGATGGACCATCTGTGTGGAGGAGCTGGGCCAGAGCGTT
AF470619        GGTGTAAATGGAAGCACGACTGGATGGACCATCTGTGTGGAGGAGTTGGGTCAGAGTGTT

ApP-349889      GTGAGTTCTTTGTGTTTGCGAGTGTTGATGT------GTGTTGAATTGCATGCTGACGAT
ApS-3372        GTGAGTCCACGTTCT-----TGTCTAGCGCATTTCGTCTTGACAGCTGCT----GACCAT
ApN-53761       GTAAGTTTTGACTTG------ATGTTGTGGA----TGATTGAGA-ATGCAGACTGACATG
ApM-64747       GTAAGTTCTGTTA--------GTGCTGTGGA----TGCTAAGGAGATACGTACTAACGAG
AF470619        GTAAGTTCTGCTT--------GTGTTGTAGA----TGCTCAAGAGATGCGTACTAACGAG

ApP-349889      T-ATACAGATTGAGTGGAAGGGCACTGATGAGGGGTGTACGCAAACCTACATTCAGGCCG
ApS-3372        -GAGATAGATCGAATGGAAGGGCACAAAGGAGGGCTGCACCCAGACCTACATTCAGGCAG
ApN-53761       AAACGTAGATTGAGTGGAAGGGCACTGATGAGGGATGCACGCAGACCTACATCCAGGCTG
ApM-64747       G-AGATAGATTGAGTGGCAGGGCACAGATGAGGGATGCACGCAGACCTACATCCAGGCTG
AF470619        G-AGATAGATCGAGTGGCAGGGCACAGATGAGGGATGCACACGGACCTACATCCAGGCTG

ApP-349889      CTTTGACCGTGCCTTATTAGGCTGACTGCTTCCTCTACGACAATCACGACATTTTCGATA
ApS-3372        CCCTGAACGTACCTTACTAGAC-GTCCGCTTTTCCGACGAGAGTTACGACCTATCAAGAT
ApN-53761       CGTTGACTGTGCCTTACTAAAA-G--TCTTGTCTGAACAAGAATCACGACGAGTCGGATA
ApM-64747       CTTTGACCAAGCCTTACTAGAT-CTCTCTAGTTTGAACGAGAATTACGACACTTCGGGTA
AF470619        CTTTGACCAAGCCTTACTAGAT-CTCTCTAGTTCAAACGAGAGTCCCGACACATCAAGTA

ApP-349889      CAACAGGGTCCGCTGGGACGGGATGAGATCACACACGAATTTGGTATCTGGAGGGAGG--
ApS-3372        CA-TTGGGTCTACTAGGACACAATTCAAACGCATACGACGTCGGCGAT-GGGAAAAGGGC
ApN-53761       CA-TCGTGTCCGCCAGAACAACACAGTG----------------------GTTTCAGAGT
ApM-64747       CA-TTGGGTCCACGAGGACAAGACGCAGGT----A------TGACAATGGGGATCAGGGC
AF470619        CA-TCGGGTCCACGAGGACAACACGCAGGC----A------TGACAATG-GAATCAGGGC

ApP-349889      A-------TCGAGCATATGCAAATCATGACAAGGATAGCCAGGCAATTATGAATCTTGCC
ApS-3372        AACCTACTTTGAACAT--TAGGATTATGG-CAAGGTAGCCAGGTAAAGAGTAACATAGCG
ApN-53761       AGCCAAAGGGGAGCAT--TTTTATGACGA-AGAGAGAGCCAGGCAGTCATGAGCCTTGTC
ApM-64747       AACCTGCTGGGAGCAT--TCGGACTAT-T-GGGGTAAAGCATGCAATCATGAACCTTGGC
AF470619        AACCTGCTGGGAGCAT--GCATACTAT-T-CTGGGAAACTAGGCAATCATGAATCTTGCC

ApP-349889      CTGGCTGGGAGGGAAGAGACACTGTAACAACATCGAACAACCA----A-TAAATTCCTAC
ApS-3372        G-ATCAGA-----GCAGAACACTGTGAT-------AACAGGTATCATA------------
ApN-53761       T-GGCAGAT---GGTTAAGCAATGTAATAATA-CTAGCAGGCATACATTAGTAACCTAAC
ApM-64747       T-GGCGGAG---GAATAGGCACTGTAACACTA-CTAGCATATATATCACGTTATTTCATC
AF470619        T-AGCGGGA---AAATGGGCACTGTAACACTA-CTAGCAAATATATCATGTTATCTCAAC

ApP-349889      TACAAA---CTTTCCCCTTTGATATAATAAGCCGTCTCA-------TGGGTACCATACTT
ApS-3372        ------CA-------------------TGAATCAACGT----------GGGG-CTTTCTA
ApN-53761       CAC-CTAATCGTACTCCAC-AATCT--CGTGT-------------------G-CTTATCA
ApM-64747       CACACTCTTCTTTCTTCTT-GTT----CATGTCCTCGATGATGGTCAGGATG-ATCATT-
AF470619        CCC-CTCTTTATTCTTCTT-GAT----AGAGTCTTTGGAAAA-----------ATCAACA

ApP-349889      TCAAACCACGTACCA--CCTCCA---------------------------TCTATCGGGC
ApS-3372        CCAAT--GC----TG-----TAATGTGTGTAAATGTGAGGAACGTGTCGTAATCACGGA-
ApN-53761       CCTTT--TCGTACAGGTGTTGCTTGTGTGCAAGTGGAATTGGCTTGAG--AATCTGGCAA
ApM-64747       TCACT--TCGAGCTGTACATACCA-----------------AGCTAAAA--GTTCAAGTGA
AF470619        CCGTT--TCGAGATA---ATGCCT----------------A------T--GTTCTAGCTC

ApP-349889      TTTCTTCTGTGCCGCACACCTTCGCAGAGAAGCAAC---------TCGATC---------
ApS-3372        ATTCTTCTG-----AA------------GTTGC------------TCGTACGGGAAG-TT
ApN-53761       AGTCT----------------------------------------------TGGTTGGAC
ApM-64747       AGCCTTAAGATTTGAAATA-----ACATGAAGCATGATGATGGTGTCAAACTTTTGGAC
AF470619        AGTAT-------------------CC---AAGCA-------AGTTTCGATCTGGGTGGAG

ApP-349889      ----------ATCGCGAC-----------------AATAGCATCGACATCCAAGATATAC
ApS-3372        AGC----AACAT-ACAGCACGCGGCTATCATGTAGTGAGACTGTGAGTATCAGGATATAC
ApN-53761       TACAAGATTCGTTGCAGCAACCGGATTACCGAATTTAACGCTTCGTAGACCTC-------
ApM-64747       ATCC---TTCACACA-G-ATCCG----------------TACCTTGAATACCAA-------
AF470619        AAAC---TACACCAAAGCAACCATAATGTC---------AACGTTTAATACCAT-------

ApP-349889      TGTACAAAAATTCCAAACTCCGACATTCAATCAATGTATATTCCGTCATGTAATAAAATG
ApS-3372        ATGCCA---------TCCTACTTG----G-TCAAAGG-T----------ATGATATACTG
ApN-53761       AGGCCT----------TATGCTGAGTTCG-ATGAAGAGGA---CATT----GATAAAACG
ApM-64747       AGAGTA----------TC------------TCAAAGAGTT---CTTCGGGCGATGAACCA
AF470619        TGACTA----------TA------------TTT---GGTA---TACCAAGAGATATA--T

ApP-349889      -----TAGATTGACAC---------------CGAAACTCCTAGCCTATGACAATGAAAAA
ApS-3372        CACAGATCAGCTACTCGG------TG----CGTTGAACAATGCTTACGTCT-------GT
ApN-53761       T----AGAAGCAACGAAGATGATGTGGAAAAAGATATATCGTTGATGGCCTTG---CAA
ApM-64747       C----TTGTGTAAC----------------TGAGATACCTCGTCAATGGCTCTT---CGA
AF470619        C----ATGATCAACT--------------TTGTGTA---------ACCGCTCCT---CAT

ApP-349889      GAGACAGAAGATAGAAACAGAAAACAGAAAACATCCCACATCTCTCCCCTCCTAACCAAC
ApS-3372        GGGATTGAAGAGACA----------ATTGGCCA--------T------CTTGTTGCCA--
ApN-53761       AGGCCTGTCAAGTGGAT---AACTTTGTTGACTTCCTGCTTC------TTTGTTGCCAAA
ApM-64747       AGGCTTGATGAGGGACC---GACCCTGTCGCCATCCGTCAGC------TTCGTCTCCAAC
AF470619        AAGTTTGGTGAGAGAAC---AACCCTGTCACCTTTCATCGAG------ATCGTCTCCAAC
```
