# Supplementary figures and images for: Genome sequencing of four Aureobasidium pullulans varieties: biotechnological potential, stress tolerance, and description of new species
Source: BMC Genomics. 2014 Jul 1;15:549. doi: 10.1186/1471-2164-15-549 (PMC4227064; doi:10.1186/1471-2164-15-549)

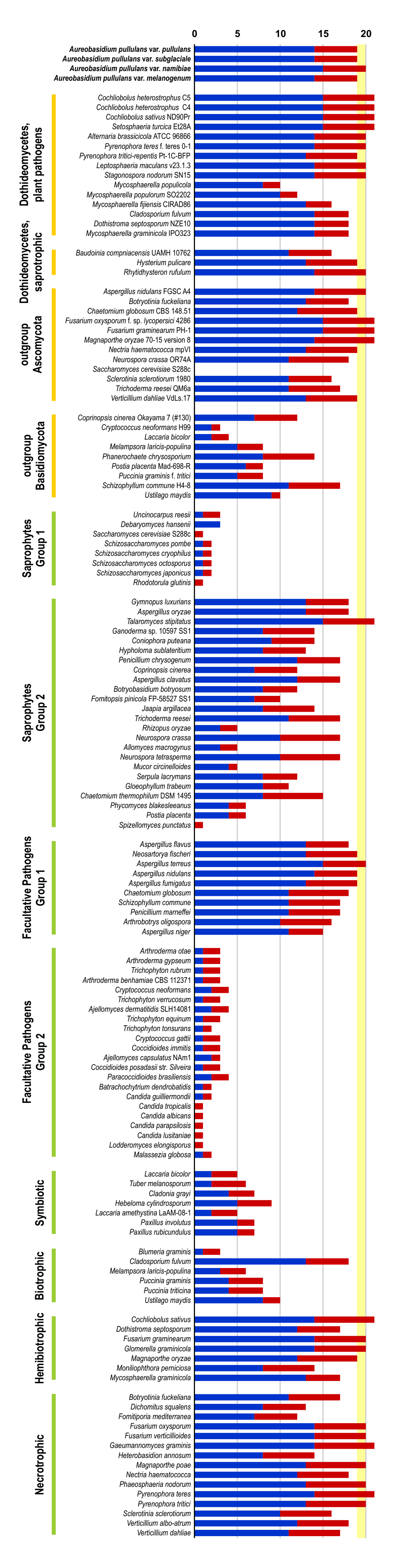

Supplement: Supplementary file 4 — Additional file 4: Different CAZy glucoside hydrolase families that are involved in degradation of plant cell walls in different fungal genomes. Blue bars, degradation of hemicellulose (GH10, GH11, GH27, GH29, GH35, GH36, GH39, GH43, GH51, GH53, GH54, GH62, GH67, GH93, GH115); red bars, degradation of cellulose (GH6, GH7, GH12, GH45, GH74, GH94, AA9 (previously GH61)); dark yellow vertical line (left), adapted from [51]; green vertical line (left), adapted from [70]. (PDF 37 KB) [file 12864_2014_7061_MOESM4_ESM.pdf]

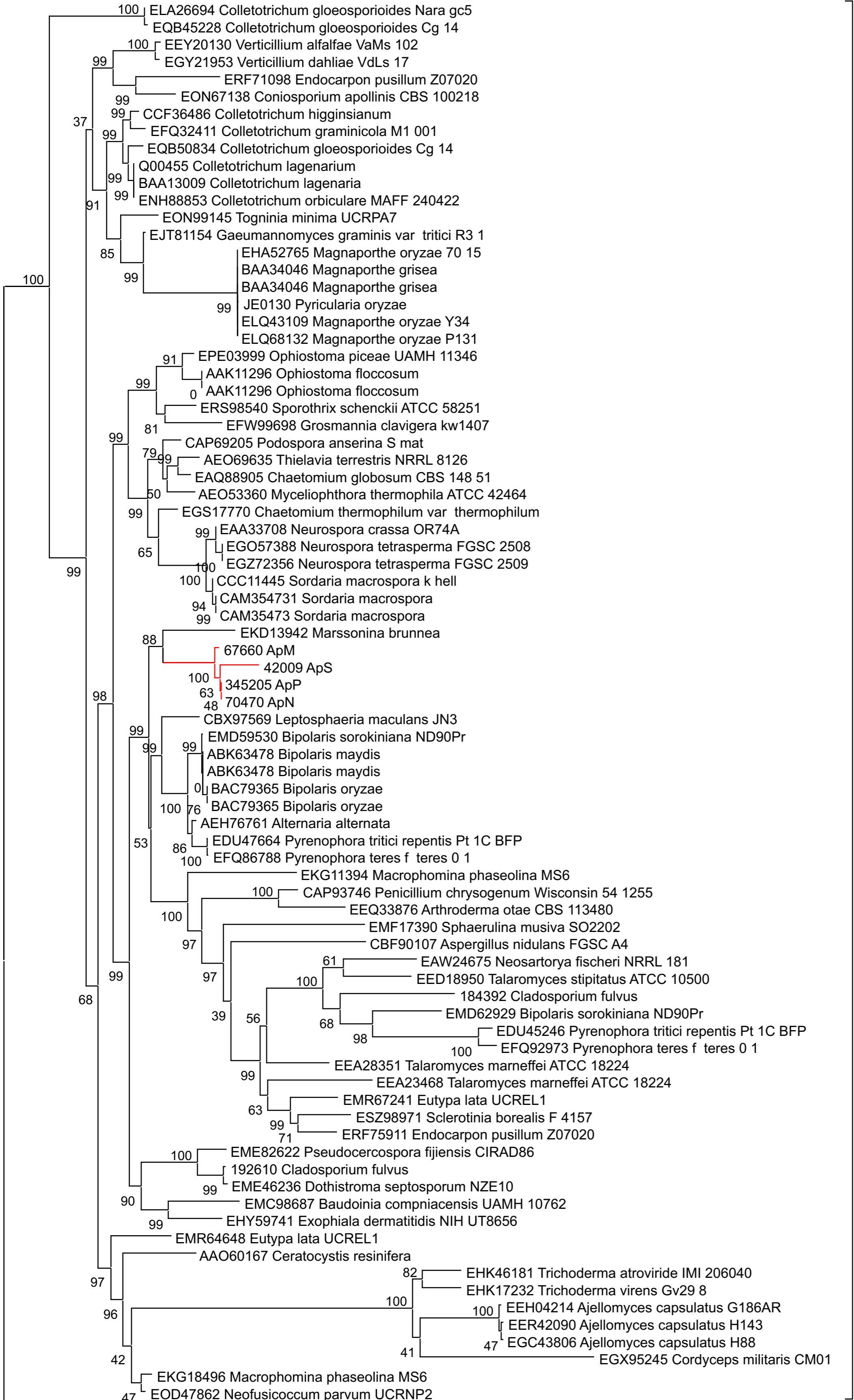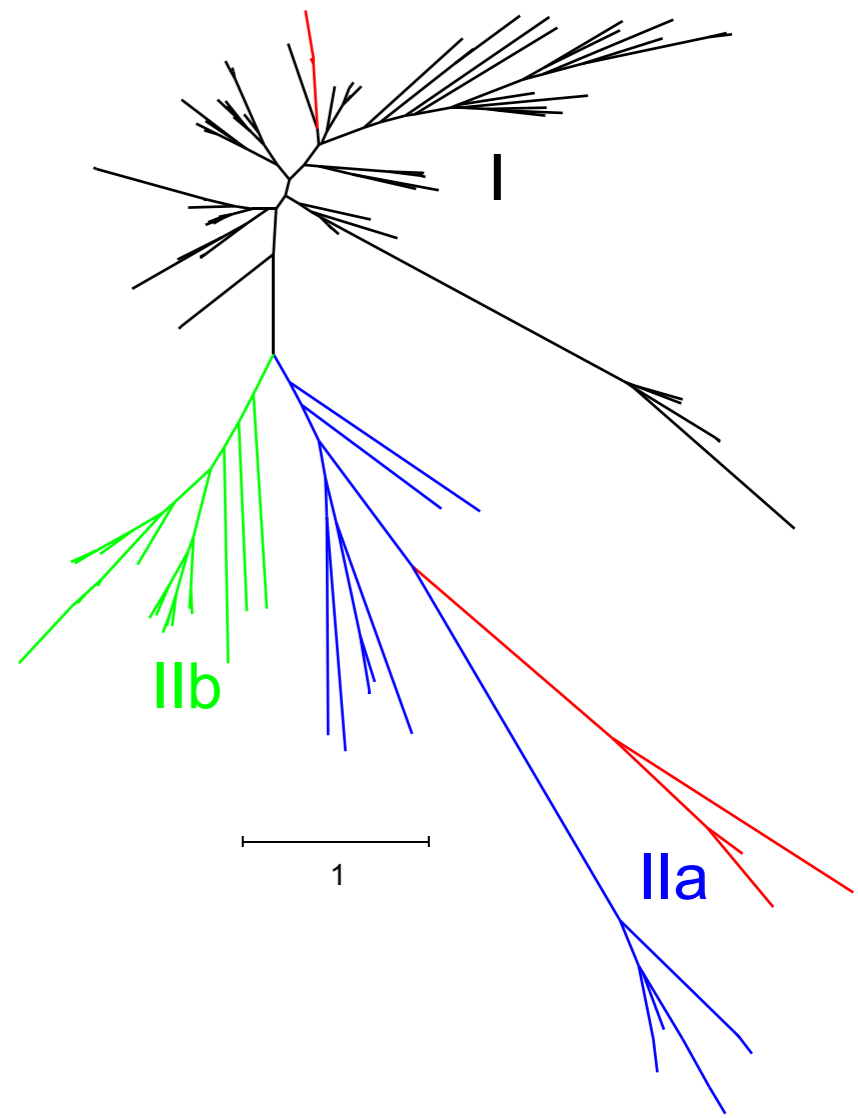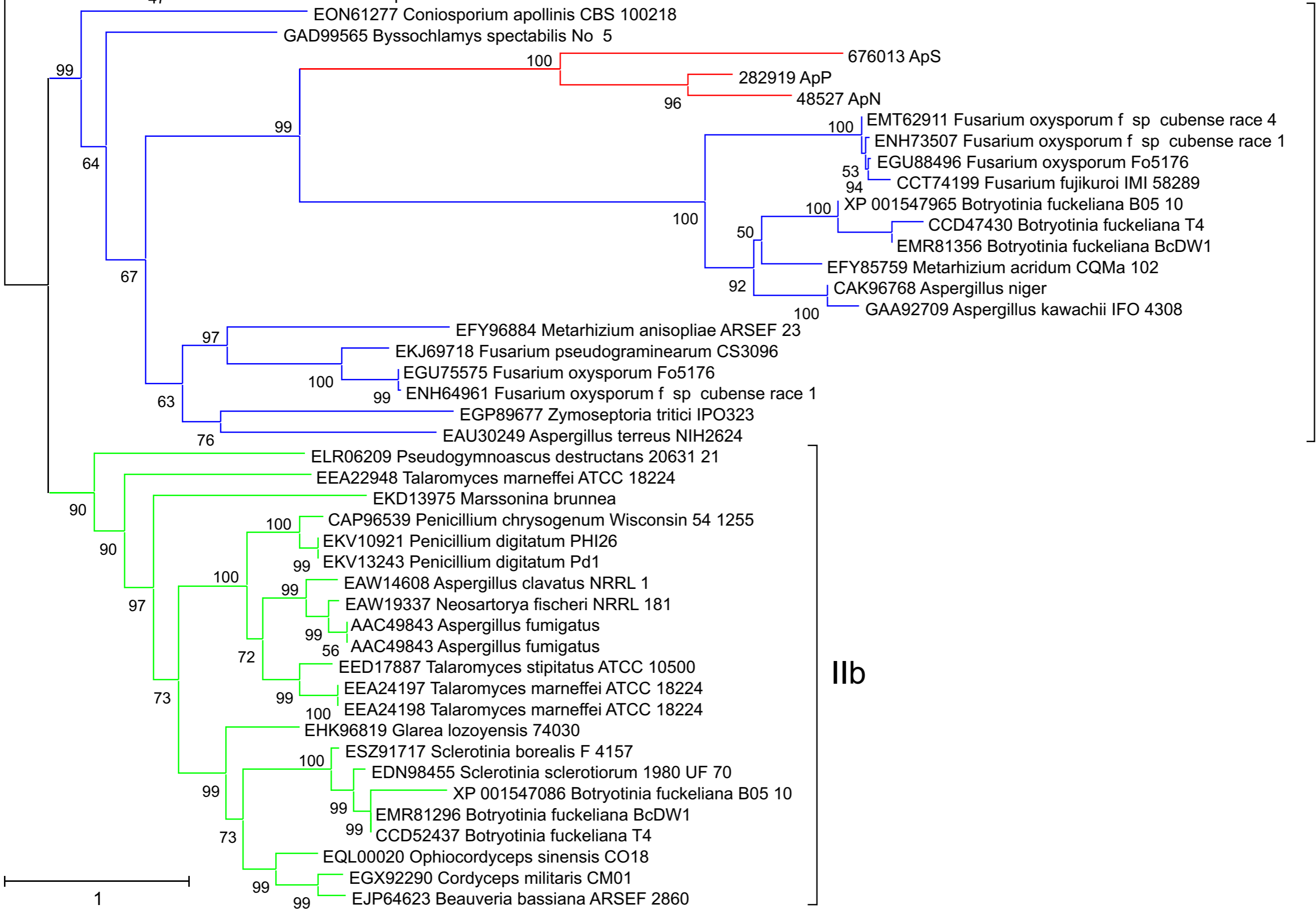

Supplement: Supplementary file 5 — Additional file 5: Protein tree of scytalone dehydratases. GenBank accession numbers of individual proteins are listed in the tree. Different gene clusters are marked with different colours. Scytalone dehydratases of the A. pullulans varieties are marked in red. (PDF 38 KB) [file 12864_2014_7061_MOESM5_ESM.pdf]

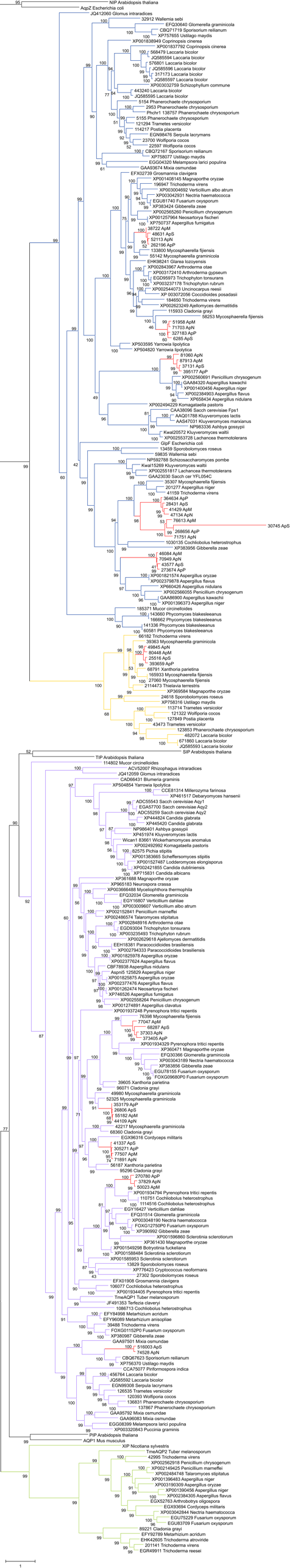

Supplement: Supplementary file 6 — Additional file 6: Protein tree of the aquaporins. The GenBank accession numbers of the individual proteins are listed in the tree. Different gene clusters are marked with different colours, which correspond to previously recognised phylogenetic groups [116]. Aquaporins of A. pullulans varieties are marked in red. (PDF 55 KB) [file 12864_2014_7061_MOESM6_ESM.pdf]

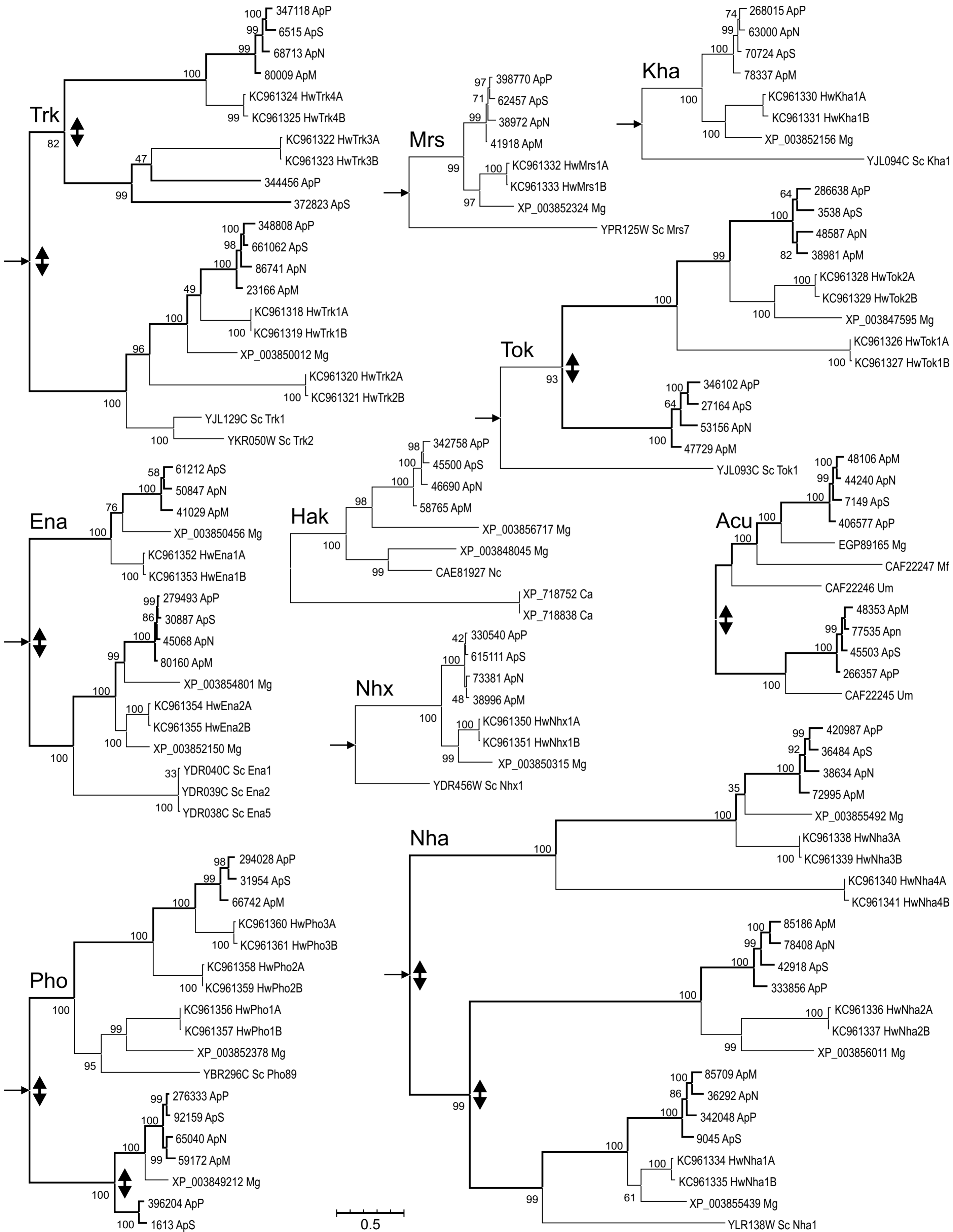

Supplement: Supplementary file 7 — Additional file 7s: Protein trees of various membrane transporters of Na + and K + . Protein trees marked with the names of the S. cerevisiae homologues (except for Acu and Hak K+ transporters, which are not found in S. cerevisiae). The trees (except for Acu and Hak) were rooted with homologous proteins from C. neoformans (Trk: [GenBank:XP_570017] and [GenBank:XP_569339]; Tok: [GenBank:XP_568987] and [GenBank:XP_568988]; Nha: [GenBank:XP_569560]; Ena: [GenBank:XP_572412], [GenBank:XP_568029] and [GenBank:XP_570160]; Pho: [GenBank:XP_568082]; Nhx: [GenBank:XP_570596]; Kha: [GenBank:XP_571501]; Vnx: [GenBank:XP_569752]; Mrs: [GenBank:XP_569566]; Pma: [GenBank:XP_568571]) and the root location is marked with an arrow. In addition to genes from A. pullulans, homologues from the following fungi were used: H. werneckii (Hw), M. graminicola (Mg) and S. cerevisiae (Sc). For Acu, homologues from Ustilago maydis (Um) and Millerozyma farinosa (Mf; Pichia farinosa) were used; for Hak, homologues from N. crassa (Nc) and C. albicans (Ca) were used. The GenBank accession numbers of the individual proteins are listed in the trees. Putative gene duplications leading to the present diversity of these genes in A. pullulans are marked with double arrows. ApP, A. pullulans var. pullulans; ApS, A. pullulans var. subglaciale; ApN, A. pullulans var. namibiae; ApM, A. pullulans var. melanogenum. (PDF 35 KB) [file 12864_2014_7061_MOESM7_ESM.pdf]

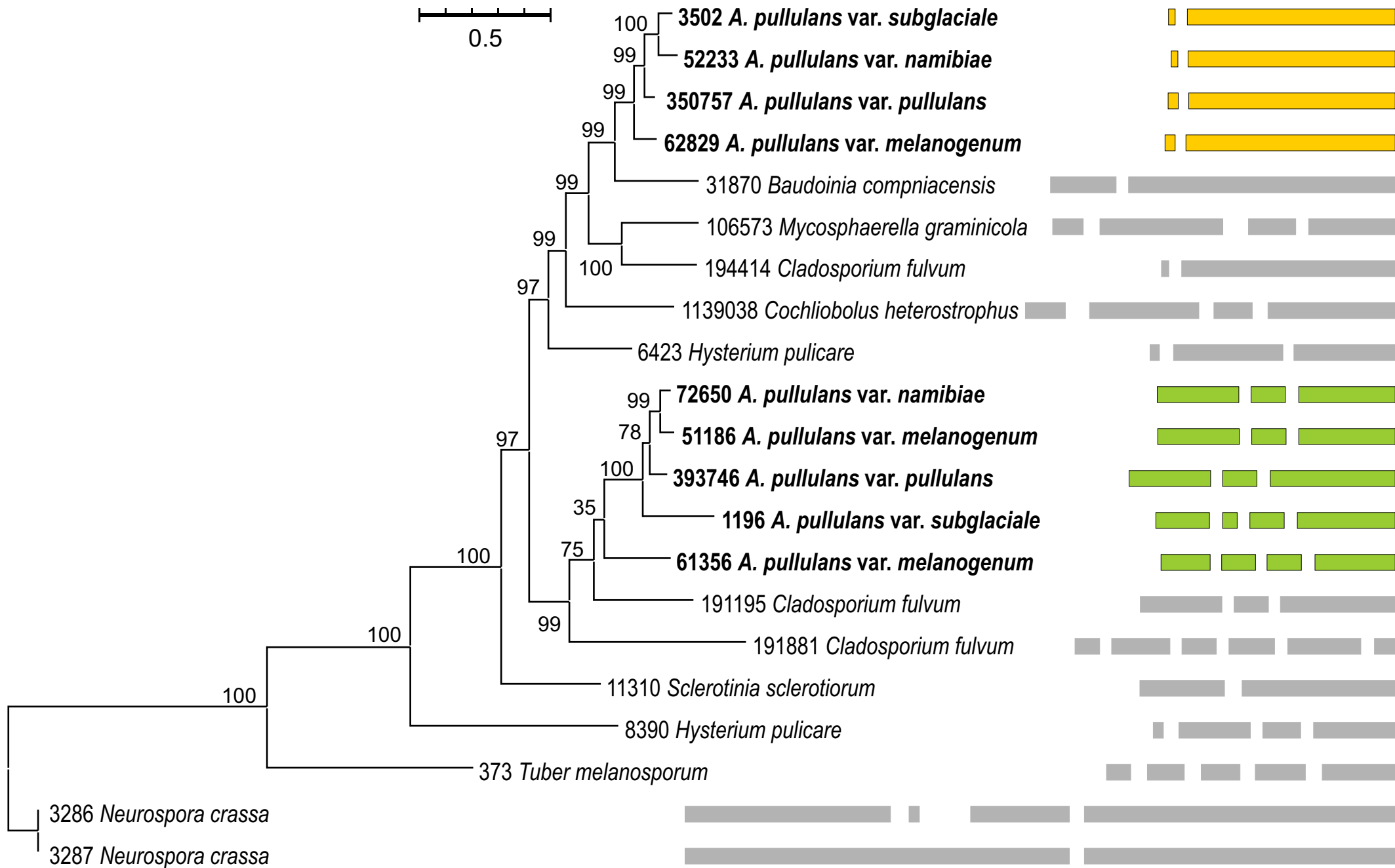

Supplement: Supplementary file 9 — Additional file 9: Phylogenetic analysis of bacteriorhodopsins from the four Aureobasidium pullulans varieties and related fungi. Numbers before species names are protein ID numbers of the Joint Genome Institute Genome Portal. The structures of the genes are represented by horizontal bars (exons) and spaces between them (introns). Lengths of the bars are proportional to the lengths of the feature represented. (PDF 22 KB) [file 12864_2014_7061_MOESM9_ESM.pdf]

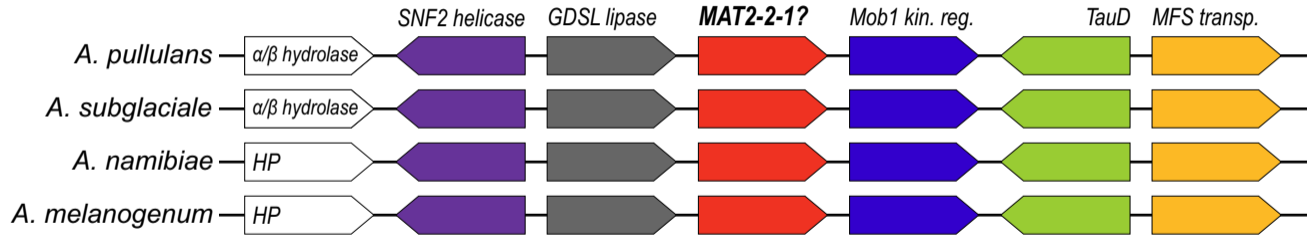

Supplement: Supplementary file 12 — Additional file 12: Putative second mating type locus, MAT2-2. Putative MAT2-2-1 genes with clearly recognisable HMG box domains (PF00505). Downstream of the putative MAT2-2-1 there is a gene with the Mob1/phocein family (Mob1 kin. reg.; PF03637), and taurine catabolism dioxygenase (TauD; PF02668), and a gene with the major facilitator superfamily (MFS_1; PF07690) domain. Upstream of the putative MAT2-2-1 there are two genes, the first encoding a hypothetical protein most similar to GDSL lipase (with no Pfam domain), and the second encoding SNF-2 helicase (PF00176). (PDF 16 KB) [file 12864_2014_7061_MOESM12_ESM.pdf]
