# Supplementary material for: Genome sequencing of four Aureobasidium pullulans varieties: biotechnological potential, stress tolerance, and description of new species
Source: BMC Genomics. 2014 Jul 1;15:549. doi: 10.1186/1471-2164-15-549 (PMC4227064; doi:10.1186/1471-2164-15-549)
Supplement: Supplementary file 11 — Additional file 11: Dot-plot comparison of Aureobasidium species scaffolds longer than 200 kbp. Six-frame translations of scaffolds aligned with Mummer 3.23. Homologous regions are plotted as dots. Scaffolds of each species are displayed ordered by decreasing size along the X and Y axes. Diagonal lines of dots in individual boxes represent syntenic regions. (PDF 7 MB) [file 12864_2014_7061_MOESM11_ESM.pdf]

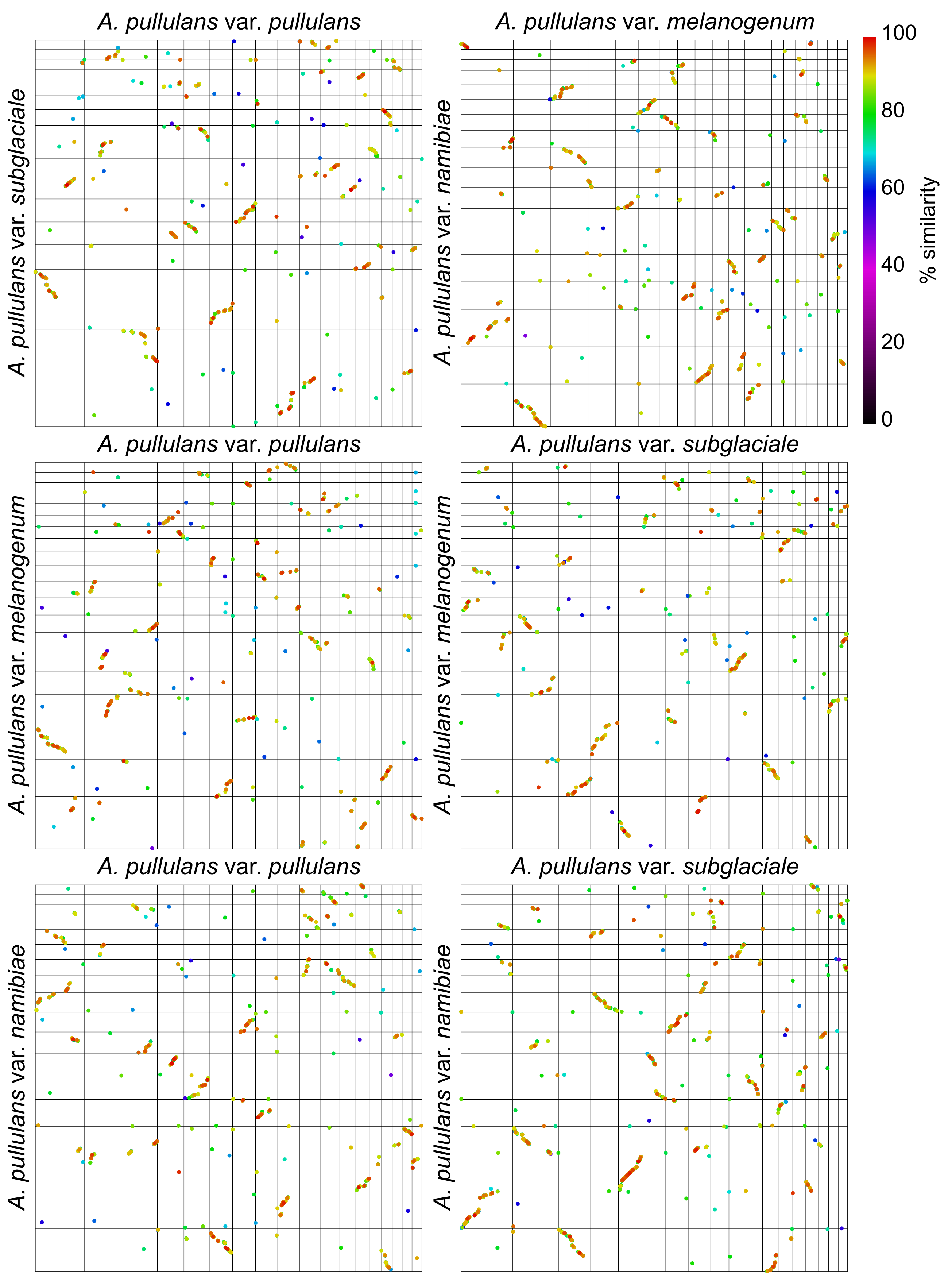
A. pullulans var. pullulans
A. pullulans var. melanogenum
A. pullulans var. subglaciale
A. pullulans var. namibiae
A. pullulans var. pullulans
A. pullulans var. subglaciale
A. pullulans var. melanogenum
A. pullulans var. melanogenum
A. pullulans var. pullulans
A. pullulans var. subglaciale
A. pullulans var. namibiae
A. pullulans var. namibiae
100
80
60
40
20
0
% similarity
